# Gender differences in body dissatisfaction: A large-scale investigation among adolescents using two international surveys

**DOI:** 10.1371/journal.pone.0330766

**Published:** 2026-03-06

**Authors:** Clotilde Napp

**Affiliations:** 1 CNRS, UMR7088, Paris, France; 2 Université Paris-Dauphine, PSL Research University, Paris, France; PLoS ONE, UNITED STATES OF AMERICA

## Abstract

Body dissatisfaction is closely linked to low self-esteem, reduced well-being, as well as mental health issues, and eating-related pathologies, which are increasingly prevalent worldwide, particularly among women. Understanding gender differences in body dissatisfaction is therefore an important issue. Yet most prior studies rely on small, non-representative samples from single developed countries. This study’s overarching goal is to provide a cross-national assessment of the magnitude and potential drivers of these gender differences. We analyze gender differences in body dissatisfaction using two large-scale international surveys, covering over 70,000 teenagers aged 15–16 across 9 countries and over 220,000 teenagers aged 11–16 across 41 countries. Girls report significantly higher body dissatisfaction than boys, regardless of Body Mass Index, socioeconomic background, age, or country. Moreover, body dissatisfaction appears more central for girls, showing stronger negative associations with life satisfaction and self-efficacy. Within countries, the gender gap is larger among high-performing students, teenagers from higher socioeconomic backgrounds, older teenagers, and those with higher BMI. The size of the gender gap varies across countries, driven mainly by variability in girls’ body dissatisfaction. Countries with larger gender gaps also show wider gender disparities in life satisfaction, eating disorders, and depression. Notably, body dissatisfaction satisfies the Gender Equality Paradox, with larger gender differences in more developed countries, mainly due to higher dissatisfaction among girls. We also examine the role of social norms. Stereotypes associating women more with physical appearance than abilities are stronger in developed countries. These stereotypes are linked to higher female body dissatisfaction and larger gender differences, and may partly explain the Gender Equality Paradox. This study identifies patterns and potential drivers of gender differences in body dissatisfaction using representative, cross-national data combined with societal indicators, and provides a foundation for more effective interventions.

## Introduction

Body dissatisfaction has been indicated repeatedly to be a major risk factor for physical and psychological health issues, including eating disorders and depression [1–7].

The prevalence of eating-related pathologies —encompassing anorexia nervosa, bulimia nervosa, and subclinical disordered-eating behaviors (e.g., fasting or laxative misuse)—has dramatically increased in the past 30 years, and primarily among women. Estimates of prevalence rates vary across studies depending in particular upon the adopted definition, but the meta-analysis [8] shows for instance prevalence rates for women that are more than twice as high as those of boys and an increase of prevalence from 3.5% for the 2000–2006 period to 4.9% for 2007–2012 and 7.8% for the 2013–2018 period. This is all the more concerning as anorexia nervosa has one of the highest mortality rates of any mental illness. Concerning depression symptomatology, its prevalence is also much higher for women than for men. Body dissatisfaction has also been related to lower self-esteem and well-being, particularly among women [9].

A better understanding of body dissatisfaction and of its differences by gender, and identification of factors that may contribute to or maintain body dissatisfaction, particularly in women, represent an important area of inquiry with significant implications for intervention.

From a theoretical point of view, objectification theory [10] and the tripartite influence model [11] have provided a framework to understand body dissatisfaction, and its relation to eating disorders and mental health issues. According to objectification theory, women are constantly looked at, evaluated, and objectified, which makes women and girls come to see themselves as an object for others to evaluate based on their appearance. They learn to internalize an observer’s view of their own body, and to evaluate it relative to prevailing social ideals (self-objectification). This experience of objectification results in body monitoring, body dissatisfaction and shame, and may engender increased anxiety, depression and disordered eating [12–16]. The tripartite influence model moreover suggests that the cultural pressure of media, parents, and peers, through the processes of social comparison and internalization of appearance ideals, shape a person’s body dissatisfaction, leading to eating disorders and psychological issues.

From an empirical point of view, recent literature concludes that girls and women report higher rates of body dissatisfaction than do men and boys [17–22]. However, significant methodological limitations constrain our understanding of these patterns. Studies have mostly relied on small, non-representative samples, often from single developed countries. In the review of epidemiology of body dissatisfaction by Frederick et al. [18] as well as in [17], it is deplored that no true nationally representative samples of body dissatisfaction currently exist. As Tylka and Hill [16] underline, empirical studies have predominantly relied on white college students from the US and Australia, with limited investigation of diverse samples or background variables such as cultural influence, socioeconomic status, etc. Moreover, inconsistent definitions of body dissatisfaction across studies [17] complicate comparisons and systematic reviews. These limitations leave unanswered the broader question of how gender differences in body dissatisfaction vary across countries and what societal factors may be related to them. To better identify and understand gender differences in body concerns —including their variation across countries and subgroups, as well as their potential determinants and implications— it is necessary to rely on larger, representative samples with more diverse populations from multiple countries that include comprehensive individual-level data. The present study directly addresses this gap by analyzing large, representative cross-national datasets.

**In this work**, we propose to analyze gender differences in body dissatisfaction based on large representative samples of teenagers between 10 and 16 y.o. observed in two different international data sources: Health Behaviour in School-Aged Children (HBSC) and Program for International Student Assessment (PISA) in 2018. Since eating disorders and most other mental health risks we have mentioned typically first emerge in late childhood or early adulthood, the consideration of adolescents seems relevant. Moreover, the samples are representative, large (over 70,000 for PISA and over 220,000 for HBSC), and cover a large number of countries, including more and less developed countries, and this is especially the case for HBSC (9 countries for PISA, 41 countries for HBSC). These two international surveys make it possible to quantify the level of body dissatisfaction for girls and boys, and the level of the gender gap, for various conceptualizations of body dissatisfaction. Moreover, these surveys also provide data on various individual characteristics, such as Body Mass Index (BMI), age, socioeconomic status, academic performance, self-esteem, and well-being, enabling an analysis of how body dissatisfaction correlates with these factors and the possible discrepancies by gender.

The cross-cultural surveys permit to obtain measures of boys’ and girls’ body dissatisfaction as well as the gender gap at the country level, and to investigate how these measures vary across countries. The variation across countries is important to investigate first because dominant theories propose that sociocultural ideals and pressures, which fluctuate across countries, play an important role in the development of body dissatisfaction and its differences by gender [11,12,23]. Variations by country are also important to investigate in relation to the Gender Equality Paradox (GEP). The GEP refers to the finding that, in some domains, larger gender differences are observed in more gender-equal and developed countries (see [24] for a recent survey). The GEP has been documented for depression [25–27], and for subjective well-being and life satisfaction [28–32]. Our two international surveys allow us to examine (i) whether gender gaps in body dissatisfaction vary across countries reflecting different cultural contexts, (ii) whether gender gaps in body dissatisfaction and gender gaps in depression and life satisfaction are related across countries, and (iii) whether the GEP also holds for body dissatisfaction—i.e., whether gender gaps tend to be larger in more developed and gender-equal countries. The GEP is highly debated and the mechanisms proposed to explain it are diverse. Some accounts emphasize methodological or measurement issues [33,34]. Other accounts, relying on evolutionary arguments, suggest that gender-equal and affluent contexts may provide greater freedom to express intrinsic gender differences [35–37]. Sociocultural perspectives instead emphasize culturally shaped processes—such as stronger self-expression values, intensified gender norms, or the salience of gendered identities in affluent and individualistic contexts [38–40]. Our international surveys allow us to investigate the potential role of such sociocultural determinants. Note that across time, the meta-analyses conducted in [19,41] show that prior to the 70s there was no significant gender difference in body satisfaction. The gender difference became statistically significant in the 80s and increased further thereafter, although [20] reach different conclusions in their cross-temporal meta-analysis of thinness-oriented dissatisfaction.

Importantly, research has consistently underlined the influence of societal gender norms on self-objectification and body dissatisfaction [9–11,23,41,42]. Specifically, a gender norm makes women more frequently associated with their physical appearance and evaluated according to it, rather than their personal abilities, compared to men [42,43]. For example, in the United States, parents search on Google ‘Is my daughter overweight’ twice as frequently as ‘Is my son overweight’ and ‘Is my daughter ugly?’ three times as frequently as ‘Is my son ugly?’. Conversely, ‘Is my son gifted’ is searched two and a half times more frequently than ‘Is my daughter gifted’, while none of these biases are grounded in reality [44]. Such cultural gender norms should vary across countries and our analysis will allow us to examine whether gender differences in body dissatisfaction are greater in countries where women are more associated with their appearance rather than their abilities. Gender norms are however difficult to measure, especially in a cross-country context. We rely on both existing data in the literature and our own measures. Leslie and coauthors [45,46] have shown that there exists a stereotype portraying men as more brilliant than women. Building on this work, we use cross-country measures of these gender stereotypes, relying on [47], with the premise that a stronger association of men with brilliance or talent implies that women’s worth may be more closely tied to their appearance than their abilities. Similarly, we use cross-country measures of stereotypes associating men with career and women with family [48], relying on [39], a higher association of men with career possibly reinforcing the association of women with their physical appearance. We also develop our own cross-country measures of gender stereotypes, specifically focusing on those more directly related to women’s appearance, such as the associations of women with beauty or body and men with talent or strength. These measures are derived using Natural Language Processing techniques, specifically word embedding models pre-trained on large text corpora, as in [49–52]. Investigating the variations of these gender stereotypes across countries, as well as their relationship with gender gaps in body dissatisfaction, will help us better understand how societal pressures contribute to gender differences in body dissatisfaction.

Our contribution compared to existing literature is threefold: First, we consider representative and diverse samples of teenagers, using large-scale surveys that allow us to explore how the gender gap in body dissatisfaction varies with background factors such as BMI, age and socioeconomic status and how body dissatisfaction relates to life satisfaction or self-esteem for boys and girls separately. Second, the cross-country datasets enable us to examine whether countries with larger gender gaps in body dissatisfaction also have greater gender gaps in life satisfaction or depression, and importantly, in relation to the Gender Equality Paradox, whether gender gaps in body dissatisfaction are more or less pronounced in more developed countries. Third, we investigate the possible role of social gender norms that associate more women with their physical appearance than with their abilities, by examining variations in these norms across countries, and their relations with gender differences in body dissatisfaction.

## Materials and methods

Materials and methods are described in detail in the Supporting Information (S1 File).

We draw on two complementary datasets, PISA and HBSC, which together provide both conceptual depth and broad cross-national coverage. PISA offers rich measures of body- and appearance-related concerns, but is restricted to 15-year-olds and to 9 countries in our sample (N ~ 70,000). HBSC, by contrast, includes a single item focused on fat-related concerns, but provides substantially broader cross-national coverage and larger samples across 41 countries (N ~ 220,000) and three age groups (11, 13, and 15 years), enabling more informative country-level comparisons. We report all results for both datasets. The consistency of results across these two datasets—despite differences in measurement approaches, country coverage, and age range—strengthens confidence in the robustness and cross-national generalizability of our findings.

### PISA2018

The first dataset is from the 2018 Program for International Student Assessment (PISA), an international assessment conducted every-3-year that evaluates the knowledge and skills of 15-year-old students in mathematics, reading, and science. PISA2018 includes a compulsory background questionnaire with information on students themselves and their household and in particular on their socioeconomic background, their life satisfaction, their negative and positive feelings, meaning in life and self-efficacy (see details in the SI). PISA2018 also includes an optional well-being questionnaire, administered in 9 countries, that addresses students’ satisfaction with various aspects of their lives, including their health, and, crucially for our analysis, their body image and appearance (via 6 specific questions). The PISA 2018 survey was administered in schools under standardized conditions. Participants were 15-year-old students enrolled in secondary education, selected through a two-stage stratified sampling design (schools were randomly sampled within each country, and students were then randomly selected within schools). This design ensures that the resulting sample is nationally representative of 15-year-old students in each participating country. The sample of teenagers who completed the well-being questionnaire in 2018 includes 71,769 observations across 9 countries (see the list in SI) with complete information on body satisfaction. The PISA well-being questionnaire includes questions about students’ height and weight, allowing for the calculation of individual Body Mass Index (BMI) based on self-reported data.

#### Measures of body dissatisfaction.

Our primary measure of body dissatisfaction (*BD*) is the (reverse-coded) body image index constructed by PISA, relying on students’ agreement with the five following statements: ‘I like my look just the way it is’ (WB153q01ha), ‘I consider myself to be attractive’ (WB153q02ha), ‘I am not concerned about my weight’ (WB153q03ha), ‘I like my body’ (WB153q04ha), ‘I like the way my clothes fit me’ (WB153q05ha). Answers are given on a four-point Likert scale with response categories ranging from “Strongly disagree”, “Disagree”, “Agree”, to “Strongly agree”. An additional response option “I don’t have an opinion” was treated as missing.

For robustness and reliability, we also consider alternative measures of body dissatisfaction by analyzing specific forms of body dissatisfaction: the *body weight concern or dissatisfaction*, measured by the reverse-coded item ‘I am not concerned about my weight’ (WB153q03ha), the *general body dislike or dissatisfaction*, measured by the reverse-coded item ‘I like my body’ (WB153q04ha) and the *body look dissatisfaction* measured by the reverse coded item ‘how satisfied are you about the way you look’ (WB155q02ha). Additionally, we consider the *weight and appearance dissatisfaction index*, which is the equally weighted average of these three measures. We systematically verify that the results obtained using our main *BD* measure are robust when using these four alternative measures.

### HBSC2018

The second dataset is from the 2018 Health Behavior in School-Aged Children (HBSC) survey, conducted by the World Health Organization (WHO) and its regional offices. This survey collects data on the health, well-being, and social environments of 11-, 13- and 15-year-old boys and girls (see details in the SI). The HBSC 2018 survey was conducted through self-administered questionnaires completed by students in classroom settings. Participants were school-aged children enrolled in regular schools at the target ages of 11, 13, and 15 years. Within each participating country, schools and classes were randomly selected using a standardized cluster sampling design, ensuring that the samples are nationally representative of children in these age groups. The HBSC survey includes information on body satisfaction (through one question only) for a total of 229,327 children across 41 different countries (see the list in SI). It also gathers data on (self-reported) body mass index, family affluence and life satisfaction.

Unlike PISA, the HBSC survey does not allow for an examination of different conceptualizations of body dissatisfaction, nor does it enable an analysis of the link between body dissatisfaction and self-efficacy or academic performance. However, it offers the advantage of covering a much larger sample with a broader age range and, importantly, providing data from 41 countries. While we report all results for both datasets, we primarily use the PISA survey for individual-level analyses and the HBSC survey for country-level analyses.

#### Measures of body dissatisfaction.

HBSC2018 includes a single question related to body image and appearance: “Do you think your body is a) much too thin, b) too thin, c) about right, d) too fat or e) much too fat?” Compared to PISA, the HBSC survey provides much less information about participants’ feelings regarding their body and appearance, as it is limited to body weight dissatisfaction. However, dissatisfaction with weight is one of the most common sources of body dissatisfaction [18] and the most directly related with eating disorders and other health issues. We focus on fatness issues, and we consider as our main measure of body dissatisfaction the *ThinkTooFat* measure, which is equal to 1 if participants answered “too fat” (d), to 2 if participants answered “much too fat” (e) and to 0 otherwise.

### Other data

We complement the PISA2018 and HBSC2018 data with the following:

(i) Several country-level measures of socioeconomic development, extent of (gender) equality and individualism to analyze the GEP in body dissatisfaction. In line with the literature on the GEP, we include the Human Development Index, Gross Domestic Product, the Gender Gap Index and Hofstede measure of Individualism (see details in the SI).
(ii) Global data on eating disorder and depression to examine the cross-country relationship between boys’ and girls’ body dissatisfaction and the prevalence of these conditions.
(iii) Cross-country data on stereotypes (indirectly or directly) related to girls’ appearance to explore the relationship between gender differences in body dissatisfaction and social norms. We use data on boys’ greater talent from Napp and Breda [47], which are based on gender differences between girls’ and boys’ perceived lack of talent in PISA. These data can be interpreted as reflecting either gender stereotypes about talent or gender differences in the belief of lacking talent. We use data about the greater association of the adjective *sexy* with girls than with boys relying on the seminal study from Williams and Best [53], data about the implicit and explicit association of men with career and women with family from [39], relying on Project Implicit [48]. Finally, we introduce measures of gender stereotypes associating beauty and body to women and talent and strength to men, relying on word embedding models pre-trained on large text corpora. We adopt the same approach as in [49–52] and use publicly available word embeddings, pretrained using the fastText algorithm on the free online encyclopedia Wikipedia, to measure the relative similarity of words related to appearance vs. abilities with women vs. men. For the Beauty-Talent stereotype, we measure the higher relative similarity of the words [“beauty”] vs. [“talent”] with the words [“woman”] vs. [“man”]. Analogously, for the Beauty-Strength stereotype (or the Body-Beauty/Talent-Strength stereotype), we measure the relative similarity of the words [“beauty”] vs. [“strength”] (or [“beauty”, “body”] vs. [“talent”, “strength”]) with the words [“woman”] vs. [“man”]. Details are provided in the SI.

### Methods

Empirical analyses presented in the paper rely on individual-level regressions, with possible controls for observable characteristics as well as country-level correlations and multivariate regressions with a few competing explanatory variables.

### Ethics statement

This study relies exclusively on publicly available and anonymized data from the Programme for International Student Assessment (PISA) and the Health Behavior in School-Aged Children (HBSC) survey. Ethical approval and informed consent were obtained by the original data collectors according to the policies of these surveys. No additional ethical approval was required for this secondary analysis.

## Results

### I. Girls are more dissatisfied with their bodies than boys and Body Dissatisfaction is more central for girls than for boys

#### A. Girls are on average more dissatisfied with their bodies.

Table 1 and Table S1A in S1 File show that girls are on average more dissatisfied with their bodies, in the PISA and in the HBSC samples.

**Table 1:** Gender gaps in body dissatisfaction.

	Gap *BD* Source: PISA2018	Gap *ThinkTooFat* Source: HBSC2018
**All**	0.15^***^ [0.14,0.17]	0.19^***^ [0.18,0.2]
	N = 75,497 observations	N = 229,327 observations
	9 countries	40 countries
**BMI**		
**BMI < 18.5**	−0.02 [−0.05,0.01]	0.12^***^ [0.11,0.13]
**“Healthy” BMI**	0.22^***^ [0.2,0.24]	0.28^***^ [0.27,0.3]
**BMI > 25**	0.16^***^ [0.12,0.2]	0.34^***^ [0.3,0.38]
**AGE**		
**Low: 11 y.o.**	NA	0.06^***^ [0.05,0.07]
**Medium: 13 y.o.**	NA	0.23^***^ [0.21,0.24]
**High: 15 y.o.**	NA	0.29^***^ [0.28,0.31]
**Socioeconomic Status**		
**Lower SES**	0.11^***^ [0.09,0.13]	0.17^***^ [0.16,0.18]
**Higher SES**	0.19^***^ [0.17,0.21]	0.22^***^ [0.21,0.23]
**Academic PERFORMANCE**		
**Lower overall perf**	0.06^***^ [0.03,0.1]	NA
**Higher overall perf**	0.22^***^ [0.19,0.24]	NA
**COUNTRIES**		
**OECD countries**	0.36^***^ [0.34,0.38]	0.23^***^ [0.22,0.24]
**Non-OECD countries**	0.04^***^ [0.02,0.06]	0.14^***^ [0.13,0.15]

Data Source: PISA 2018 and HBSC2018.

Notes: The table presents the gender gap in body dissatisfaction in the entire sample and among various groups in the PISA2018 sample (column 1) and the HBSC2018 sample (column 2). In PISA (col. 1), body dissatisfaction is measured by the variable *BD*, which is the opposite of the Body Image Index constructed by PISA based on five questions, and captures participants’ dissatisfaction with their weight, appearance and body. In HBSC (col. 2), body dissatisfaction is measured by the *ThinkTooFat* variable, which is based on the single question addressing body image issues in the HBSC survey and reflects participants’ perception of being too fat. See the main text and the SI for details on the measures of body dissatisfaction. The gender gap is given by the difference between girls and boys (G-B) in the respective measures, standardized by country. BMI denotes Body Mass Index, and we categorize participants into three groups based on their BMI level. While PISA only includes 15-year-old students, HBSC includes three age categories, which we consider as separate subgroups. Socioeconomic status (SES) is measured in PISA by the ESCS index, and in HBSC by the RFAS index, with participants grouped according to whether they are above or below the average level of SES. PISA provides information on student performance in math, reading and science and we consider as subgroups the students with an overall performance (in math reading and science) above or below the average. Finally, we distinguish between OECD and non-OECD countries in each sample. 95% confidence intervals in brackets. *** p < 0.01, ** p < 0.05, * p < 0.1.

In the PISA sample, the gender gap in our main measure *BD* is equal to 0.15SD. Body dissatisfaction is significantly and consistently higher on average for girls than for boys for all the five measures we consider, with effect sizes ranging from 0.15SD to 0.25SD. In the overall sample, approximately a quarter of boys report ‘disliking their bodies’, compared to more than a third of girls, which aligns closely with proportions found in Frederick et al.’s review of meta-analyses on gender differences in body dissatisfaction [18].

Body dissatisfaction is also, on average, higher for girls than for boys in the HBSC2018 sample, with an effect size of 0.19 SD for the *ThinkTooFat* measure. In the overall sample, approximately 21% of boys and 29% of girls perceive themselves as “too fat” or “much too fat” with girls being twice as likely as boys to describe themselves as “much too fat” (4.3% vs. 2.4%).

#### B. The gender gap in body dissatisfaction is robust to controls.

We show in Table S1B in S1 File that the gender gap in body dissatisfaction is robust to controls.

In the PISA sample, we show for the five different measures of body dissatisfaction the robustness to controlling for Body Mass Index (BMI), socioeconomic background, academic performance, life satisfaction, negative and positive feelings, meaning in life, self-efficacy and other factors like grade repetition or parental education level.

In the HBSC sample, we control for BMI, family affluence, age, and life satisfaction.

With all controls included, the effect size in *BD* (in PISA) is equal to 0.13SD and the effect size in *ThinkTooFat* (in HBSC) is equal to 0.21SD.

This robustness analysis shows in particular that higher body dissatisfaction among girls is not attributable to gender differences in BMI nor to girls’ higher general tendency toward negative feelings or life dissatisfaction.

#### C. Robustness and marked variability of the gender gap across subgroups.

The gender gap in body dissatisfaction could theoretically be limited to specific groups, such as individuals with high BMI, or individuals from low socioeconomic background, or given countries. However, as shown in Table 1 and Table S1C in S1 File, this is not the case. We analyze various subgroups based on BMI, socioeconomic background, academic performance (PISA only) and age (HBSC only). The gender gap in body dissatisfaction is observed across essentially all these subgroups, but it varies substantially across subgroups (Table S1Ci in S1 File).

We first consider subgroups based on Body Mass Index (PISA and HBSC). The gap is the lowest among those with a low BMI. For instance, the gender gap in the main measure *BD* in the PISA sample is 0.22SD for participants with a BMI in the ‘standard range’ (between 18.5 and 25) compared to −0.02SD for those with a lower BMI and the gender gap in *ThinkTooFat* in the HBSC sample is 0.28SD for standard BMI, compared to 0.12SD for a lower BMI. Girls with low BMI are relatively less dissatisfied with their bodies (see also Table S1Cii in S1 File).

When examining socioeconomic background (PISA and HBSC) and academic performance (PISA only), the gender gap remains significant among all subgroups. However, it tends to be more pronounced in ‘privileged’ conditions—higher socioeconomic background, and higher academic performance. For instance, in the PISA sample, the gender gap in *BD* is 0.06SD for participants with below-average overall academic performance, compared to 0.22SD for those with above-average overall academic performance.

These patterns are confirmed when we run a multiple linear regression of body dissatisfaction on gender, socioeconomic background, BMI (and performance for the PISA sample) as well as their interactions with gender: all the coefficients of these interactions are significant and positive (Table S1C in S1 File) in both the PISA and the HBSC samples.

The HBSC sample permits to consider different ages. Unlike PISA, which only includes 15-year-olds, the HBSC survey allows us to examine three age groups [11,13,15]. The gender gap is significant across all age groups, with a trend of increasing with age. Among 15-year-olds (the same age group as in PISA), the gender gap in *ThinkTooFat* is 0.29SD, whereas it is equal to 0.06SD at age 11. This variation is mainly due to girls (Table S1Cii in S1 File): girls’ *ThinkTooFat* level increases from 0.27 for the first age group, to 0.38 for the second age group and 0.4 for the third age group while boys’ *ThinkTooFat* level remains almost constant.

#### D. Body dissatisfaction is related to life satisfaction and self-efficacy, especially for girls.

In Table 2 and Table S1D in S1 File, we show that (i) body dissatisfaction is significantly related to indicators of life satisfaction, self-worth and well-being, aligning with the understanding that body dissatisfaction is closely connected to one’s perception of self-worth and overall well-being, (ii) controlling for body dissatisfaction significantly alters the estimated gender differences in life satisfaction, self-worth and well-being, suggesting that girls’ higher body dissatisfaction may partly account for gender disparities in these outcomes; and (iii) body dissatisfaction appears more “central” for girls than for boys as the relationship between body dissatisfaction and indicators of life satisfaction, self-worth and well-being is stronger for girls than for boys.

**Table 2:** Differential impact of body dissatisfaction on life satisfaction or negative feelings for boys and for girls.

	*Dependent Variable is...*		
	LIFE SATISFACTION	LIFE SATISFACTION	FEELING MISERABLE
	Source: HBSC	Source: PISA	Source: PISA
Girl	−0.099***	−0.089***	0.188***
	[−0.11,−0.09]	[−0.1,−0.08]	[0.17,0.2]
Body Dissatisfaction	−0.137***	−0.257***	0.160***
	[−0.14,−0.13]	[−0.27,−0.25]	[0.15,0.17]
Girl*Body Dissatisfaction	−0.092***	−0.081***	0.084***
	[−0.1,−0.08]	[−0.1,−0.07]	[0.07,0.1]
Constant	0.054***	0.046***	−0.099***
	[0.05,0.06]	[0.04,0.06]	[−0.11,−0.09]
Observations	226,024	71,313	69,518
R-squared	0.042	0.096	0.056

Data Source: PISA2018 and HBSC2018.

Notes: The table displays estimates of the regression of indicators of life satisfaction (first two columns) and of the frequency of negative feelings, specifically feeling miserable (third column), on gender, body dissatisfaction and their interaction. The first column uses the HBSC sample, while the other two columns use the PISA sample. In PISA, body dissatisfaction is measured by the variable BD, defined as the reverse of the body image index constructed by PISA; higher scores indicate greater dissatisfaction with one’s weight, appearance, and body. Life satisfaction is assessed with the question *“Overall, how satisfied are you with your life as a whole these days?”* answered on a 0–10 scale. The variable Feeling miserable is based on responses to the question *“How often do you feel miserable?”* with four response options (“never”, “rarely”, “sometimes”, “always”). In HBSC, body dissatisfaction is measured by the variable ThinkTooFat, which captures participants’ perception of being too fat, and life satisfaction is measured using a 0–10 scale, ranging from 0 (“worst possible life”) to 10 (“best possible life”). All variables are standardized to have a weighted mean of 0 and a weighted standard deviation of 1 in each country. Details on all measures can be found in the SI. 95% confidence intervals in brackets. ^*^ *p* < 0.10, ^**^ *p* < 0.05, ^***^ *p* < 0.01.

For the PISA sample, Table 2 and Table S1D in S1 File show that for both boys and girls, higher levels of body dissatisfaction are generally associated with lower life satisfaction, reduced self-efficacy, diminished meaning in life, and increased negative feelings across the entire sample. Additionally, Table S1D in S1 File further shows that controlling for body dissatisfaction alters the gender gaps in these variables. For instance, the gender gap in life satisfaction is equal to −0.14SD without control, but is reduced to −0.09SD when body dissatisfaction *BD* is controlled for, suggesting that one of the possible paths for higher life dissatisfaction of girls is their higher body dissatisfaction. Finally, body dissatisfaction is a stronger predictor of life satisfaction, negative feelings, self-efficacy, and the sense of meaning in life for girls than for boys (Table 2 and Table S1D in S1 File). For example, a 1SD increase in the measure of body dissatisfaction *BD* is associated with a 0.16SD increase in the frequency of feeling miserable for boys (with an R-squared of 0.02), whereas for girls, the same increase in *BD* corresponds to a 0.24SD increase in the frequency of feeling miserable (with an R-squared of 0.06).

The HBSC 2018 survey does not provide data on self-efficacy or negative feelings, as PISA does, but it does include information on life satisfaction. Similar to the PISA sample, body dissatisfaction predicts life dissatisfaction for both boys and girls, and controlling for body dissatisfaction reduces the gender gap in life satisfaction. Body dissatisfaction is also a stronger predictor of life dissatisfaction for girls than for boys (Table 2 and Table S1D in S1 File). For example, a 1SD increase in *ThinkTooFat* is associated with a 0.14SD decrease in life satisfaction for boys (with an R-squared of 0.02) and a 0.23SD decrease in life satisfaction for girls (with an R-squared of 0.06).

The modest R-squared values in Table 2 (and Table S1D in S1 File) reflect both the very large sample sizes and our analytic focus. We do not aim to explain life satisfaction or negative feelings comprehensively, but rather to document that body dissatisfaction is significantly associated with these outcomes, and more strongly so for girls than for boys—as evidenced by the significant negative interaction terms across all specifications.

### 2. The gender gap in body dissatisfaction across countries and the GEP in body dissatisfaction

We now examine gender differences in body dissatisfaction across countries. First, we describe the variations in the gender gap in body dissatisfaction across countries. Next, we analyze whether countries with larger gender differences in body dissatisfaction also exhibit larger gender differences in life satisfaction, eating disorders and depression. Finally, in connection with the Gender Equality Paradox, we investigate how these disparities relate to the level of development in these countries (see the Materials and Methods section for details about the measures of country development). For each analysis, we also assess whether the observed variations and relations across countries are primarily driven by girls or boys.

The small number of countries in the PISA sample (N = 9) limits the scope for robust cross-country analysis and we focus on the HBSC sample (N = 41), which includes a larger number of countries.

#### A. Robustness and variability of the gender gap in body dissatisfaction across countries, primarily driven by girls.

Table S2Ai in S1 File displays the level of the gap in Body Dissatisfaction by country. We observe that girls have a higher level of body dissatisfaction in almost all countries.

In the PISA sample, for every country, there is at least one measure of body dissatisfaction (out of the five we consider) showing a significant gender gap. In the HBSC sample, the gap in *ThinkTooFat* is significant and positive in 34/41 countries.

Although positive in most countries, the gender gap in body dissatisfaction exhibits a great variability across countries, which is largely attributable to girls’ variations across countries (Table S2Aii in S1 File).

*PISA2018 sample*. The gender gap in *BD* varies from −0.08SD in the United Arab Emirates, where the gap is negative, to as much as 0.59SD in Ireland, with a standard deviation across countries of SD = 0.21. This variation across countries is primarily driven by differences in girls’ body dissatisfaction (Table S2Aii in S1 File): The standard deviation of girls’ *BD* level across countries (0.27) is nearly twice that of boys (0.16), and girls’ *BD* shows a significant relationship with the gender gap (*r* = 0.82), which is not the case for boys (*r* = 0.16). This pattern is consistent across all five considered measures of body dissatisfaction.

*HBSC2018 sample*. At the country level, the gender gap in *ThinkTooFat* ranges from non-significant levels in countries such as Albania or Azerbaijan to approximately 0.40 SD in Belgium, the Netherlands or Sweden, with a standard deviation across countries of SD = 0.12. Consistent with the PISA sample findings, the variation in the gender gap in body dissatisfaction across countries is mainly due to girls’ (Table S2Aii in S1 File): The standard deviation is greater (0.11 vs. 0.06) and the correlation with the gender gap in body dissatisfaction is higher for girls compared to boys (0.79 vs. 0.30).

#### B. Relationship between gender gaps in body dissatisfaction and gender gaps in eating disorders, depression, and life dissatisfaction, primarily driven by girls.

Table S2Bi in S1 File shows that countries with larger gender gaps in body dissatisfaction also exhibit larger gender gaps in eating disorders, in depression, and in life satisfaction. Table S2Bii in S1 File further shows that this relation between gender gaps is primarily driven by girls.

*PISA2018 sample*. Although not all correlations are significant on this small sample (N = 9), they consistently point in the same direction.

*HBSC2018 sample*. A one standard deviation increase in the gender gap in *ThinkTooFat* is associated with an increase of 0.38SD in the gender gap in eating disorders (0.40SD for 15year-olds only; N = 41), 0.31SD in the gender gap in depression (0.34SD for 15year-olds only; N = 41) and 0.63SD in the gender gap in life satisfaction (0.59 SD for 15 year-olds only).

When broken down by gender, the relationship is primarily driven by girls’ body dissatisfaction and its association with higher levels of eating disorders, life dissatisfaction and depression among girls (Table S2Bii in S1 File). Across countries, greater body dissatisfaction in girls, as measured by *ThinkTooFat* within the HBSC sample, is significantly associated with higher levels of eating disorders (*r* = 0.4), life dissatisfaction (*r* = 0.48) and depression (*r* = 0.29). These associations are not observed for boys (or are smaller), contributing to larger gender gaps in these disorders.

#### C. Gender gaps in body dissatisfaction are higher in more gender equal and developed countries.

We first observe (Table 1 and Table S2Ai in S1 File) that in the PISA sample, the gender gap in body dissatisfaction is much larger in OECD countries (0.36SD for *BD*, 3 countries), which tend to be more economically developed, compared to non-OECD countries (0.04SD for *BD*, 6 countries), and the same pattern is true in the HBSC sample, with a gender gap in *ThinkTooFat* equal to 0.23SD among OECD countries (N = 26) and 0.14SD among non-OECD countries (N = 15).

Table 3 and Table S2C in S1 File provide the results of regressions and correlation analyses between gender gaps in body dissatisfaction and indicators of country development such as the Human Development Index (HDI) or the Gross Domestic Product (GDP). Measures of country gender equality such as the Gender Gap Index (GGI) and of individualism (Hofstede) are also considered, as they are commonly discussed in the literature on the Gender Equality Paradox.

**Table 3:** The gender equality paradox in body dissatisfaction.

	GAP	GAP	GIRLS’	GIRLS
	Body	Body	Body	Body
	Dissatisfaction	Dissatisfaction	Dissatisfaction	Dissatisfaction
**HDI**	0.618***		0.651***	
	[0.34,0.876]		[0.402,0.9]	
**lGDP**		0.575***		0.582***
		[0.31,0.84]		[0.318,0.845]
Constant	<0.001	<0.001	<0.001	<0.001
	[−0.25,0.25]	[−0.26,0.26]	[−0.25,0.25]	[−0.26,0.26]
Obs.	40	41	40	41
R-squared	0.382	0.331	0.424	0.339

Data Source for measures of Body Dissatisfaction: HBSC2018.

Notes: The table presents estimates at the country level in the HBSC sample of linear regressions of gender gaps (Girls-Boys) in body dissatisfaction (columns 1–2), and girls’ levels of body dissatisfaction (columns 3–4), measured by the variable *ThinkTooFat*, on indicators of country level of development: the Human Development Index (HDI, row 1) and the log of the Gross Domestic Product (lGDP, row2). The *ThinkTooFat* measure captures participants’ perception of being too fat. All variables and data sources are described in Appendix A in the SI. All variables are standardized on the regression sample. 95% confidence intervals in brackets. ^***^ *p < 0.01,* ^**^ *p < 0.05,* ^*^ *p < 0.1.*

*PISA2018.* The gender gap in *BD* is significantly and positively correlated with HDI (*r* = 0.66, N = 9). Similar results are observed with the GGI (*r* = 0.69) and with Hofstede’s measure of individualism (*r* = 0.83). These results are consistent across all five considered measures of body dissatisfaction. It is worth noting that the correlation with Gross Domestic Product (GDP) is weaker, but mainly due to one country, the United Arab Emirates.

**Fig 1:**
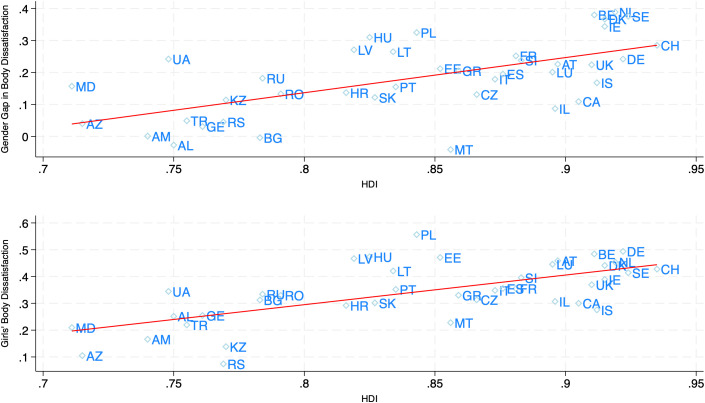
Gender gap in body dissatisfaction and girls’ level of body dissatisfaction as a function of country HDI. The figure displays the country-level gender gap (Girls-Boys) in body dissatisfaction, as well as girls’ body dissatisfaction, as a function of country level of Human Development Index (HDI). Body dissatisfaction is measured by the variable *ThinkTooFat,* which captures participants’ perception of being too fat. For the gender gap, the *ThinkTooFat* measure is standardized at the country level. Details on all measures can be found in the main text and the SI. Country codes from ISO3166-1 standard. Data Source for measures of Body Dissatisfaction: HBSC2018.

*HBSC2018.* The gender gap in *ThinkTooFat* is significantly positively correlated with HDI (*r* = 0.62, N = 41). Fig 1 (top figure) illustrates this result. Similar correlations are observed with GDP (*r* = 0.58). The relationships are also significant with the GGI (*r* = 0.47) and Hofstede’s measure of Individualism (*r* = 0.61).

To verify that the GEP is not driven by unobserved heterogeneity among teenagers from more or less developed countries (such as differences in BMI, household resources or age), Table S2D in S1 File presents estimations of individual-level regressions of body dissatisfaction on a gender dummy, and the interaction term between gender and measures of country wealth and development. Various specifications are included, incorporating controls such as BMI, household resources, age category, and life satisfaction (at the cost of reducing the sample size). Country fixed effects are included in all models. We observe that the coefficient of the interaction term between gender and the level of development or wealth of the country remains positive and significant across all specifications, with minimal variation. Notably, the relation between the gender gap in body dissatisfaction and country development and wealth is slightly reduced (from 0.074 to 0.062 for HDI) but remains significant even when controlling for individual life satisfaction, which means that the greater body dissatisfaction observed among girls compared to boys in more developed countries cannot be solely attributed to their higher (relative) dissatisfaction with life (the GEP in life satisfaction [28–32]).

Finally, Table 3 and Table S2E in S1 File indicate that the relationship between body dissatisfaction and economic development and gender equality is significant for girls, and more pronounced for girls than for boys. For example, the correlation between *ThinkTooFat* and HDI is equal to 0.65 for girls (see also Fig 1) and 0.47 for boys. This suggests that the GEP is primarily driven by increasing body dissatisfaction among girls in more economically developed countries.

### 3. Gender stereotypes

We now examine the possible role of gender stereotypes that relate, more or less directly, to the association of women with their physical appearance in explaining cross-country variations in gender differences in body dissatisfaction (see the Materials and Methods section and Appendix A in the SI for details about the considered measures of gender stereotypes).

We start by considering gender stereotypes already analyzed in previous literature such as those associating men with talent or brilliance [45–47] or with career [39]. The underlying idea is that the less women are associated with talent and career, the more likely they will be associated with physical appearance and beauty. To build on this, we incorporate data on gender stereotypes more directly related to women’s physical appearance. First, we draw on the seminal cross-national study of gender stereotypes by Williams and Best [53], which asked respondents about the stereotypical association of a standardized list of trait adjectives with women versus men in their country. We use the item *sexy* from this list to capture the extent to which the appearance and sexual attractiveness are stereotypically associated with women rather than men. Second, we use Natural Language Processing techniques, specifically word embedding models pre-trained on large text corpora (Wikipedia), to construct country-level indices of appearance-related gender stereotypes. These indices capture whether words related to beauty and physical appearance are more strongly associated with female than male words, relative to attributes such as talent or strength. More precisely, we consider the beauty vs. talent stereotype, the beauty vs. strength stereotype and the beauty/body vs. talent/strength stereotype. As outlined in [52], these measures relying on word embeddings are a useful tool for the cross-country analysis of gender stereotypes, although they are subject to noise and should be interpreted with caution.

#### A. Gender stereotypes related to physical appearance and beauty are more pronounced in more developed countries.

We first observe in Table S3Ai in S1 File that these various measures of gender stereotypes are positively and significantly related across countries, which suggests that they truly capture the strength of gender stereotypes related to the association of women with their physical appearance at the country level. Countries where girls more than boys believe lacking talent (in PISA) are those where career is more associated to men and family to women (in Project Implicit) and also those where physical appearance and beauty are more associated with girls and women (in large text corpora such as Wikipedia).

Moreover, it is shown in previous literature that gender stereotypes about talent [47] and those about career and family [39] are more pronounced in more developed countries. We show in Table S3Aii in S1 File that the association of the trait adjective *sexy* with women vs. men [53] is also stronger in more developed countries, although the sample size is limited (N = 25 countries). Notably, Table S3Aii in S1 File shows that stereotypes associating beauty and physical appearance more strongly with women than men relative to attributes like talent or strength (measured through word embeddings) are stronger in more developed countries. For instance, the beauty/body vs. talent/strength stereotype has a correlation coefficient *r* = 0.36 with HDI and *r* = 0.45 with GDP. The stronger gender stereotypes related to women’s physical appearance in more developed countries, as captured by our survey- and text-based measures, may contribute to explaining why gender differences in body dissatisfaction are greater in these countries.

#### B. Gender differences in body dissatisfaction and gender stereotypes.

We now explicitly examine the relationships between gender stereotypes and gender gaps in body dissatisfaction. Table 4 and Table S3Bi in S1 File show that greater levels of gender stereotypes are associated with larger gender differences in body dissatisfaction.

**Table 4:** Gender differences in body dissatisfaction and gender stereotypes.

	*Dependent Variable is*			
	*the Gender Gap in Body Dissatisfaction*			
	*(ThinkTooFat)*			
**Gender Talent Stereotypes**	0.731***		0.635***	
	[0.493,0.969]		[0.279,0.991]	
**Gender Beauty** **Stereotypes**		0.504***		0.352**
		[0.193,0.815]		[0.080,0.625]
**HDI**			0.130	0.513***
			[−0.226,0.486]	[0.24,0.785]
Constant	<0.001	<0.001	<0.001	<0.001
	[−0.23,0.23]	[−0.31,0.31]	[−0.24,0.24]	[−0.26,0.26]
Obs.	36	34	36	34
R-squared	0.535	0.254	0.542	0.494

Data Source for measures of Body Dissatisfaction: HBSC2018.

Notes: The table presents country-level estimates in the HBSC sample of linear regressions of gender gaps (Girls-Boys) in body dissatisfaction, measured by the variable *ThinkTooFat*, on indicators of gender stereotypes (columns 1–2) and on both an indicator of gender stereotypes and a country level of development indicator (columns 3–4). The *ThinkTooFat* measure captures participants’ dissatisfaction with their body fatness. For gender stereotypes, we consider an indicator of country-level gender stereotypes about talent or brilliance (the *Gender Talent Stereotypes* measure taken from [47], row1) and an indicator of gender stereotypes about females’ bodies and beauty, based on large text corpora introduced in this study (see main text and SI). We use the Human Development Index (HDI) as a measure of country development level. All variables and data sources are described in Appendix A in the SI. All variables are standardized on the regression sample. 95% confidence intervals in brackets. ^***^ *p < 0.01,* ^**^ *p < 0.05,* ^*^ *p < 0.1.*

*PISA sample*. Although the PISA sample includes only a limited number of countries, the results in Table S3Bi in S1 File suggest that countries with stronger association of talent to men tend to have larger gender gaps in body dissatisfaction (R = 0.79 for *BD*). For gender stereotypes associating men with career, correlations are also positive but not always significant. The same is true for measures of gender stereotypes about women’s physical appearance relying on word embeddings, but the number of observations is very small (N = 7) and one single country can strongly impact the results.

**Fig 2:**
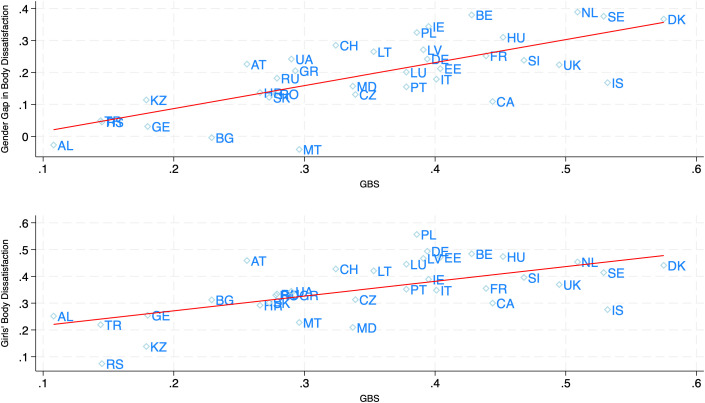
Gender gap in body dissatisfaction and Girls’ level of body dissatisfaction as a function of country Gender Brilliance Stereotypes. The figure displays the country-level gender gap (Girls-Boys) in body dissatisfaction as well as girls’ body dissatisfaction as a function of country-level Gender Brilliance (or talent) Stereotypes (GBS). Body dissatisfaction is measured by the variable *ThinkTooFat,* which captures participants’ perception of being too fat. For the gender gap, the *ThinkTooFat* measure is standardized at the country level. Country-level GBS measures are taken from [47] and are based on gender differences in attributing failure to a lack of talent in PISA2018. Details on all measures can be found in the main text and the SI. Country codes from ISO3166-1 standard.

*HBSC sample.* An increase of one SD in gender talent stereotypes is associated with a 0.73SD increase in the gender gap in *ThinkTooFat* (N = 36, see Table 4 and Table S3Bi in S1 File). Fig 2 (top figure) illustrates this result. Although quantitatively less strong, similar positive and significant relations are observed for career-family gender stereotypes as well as for beauty stereotypes relying on large text corpora. For instance, an increase of one SD in gender stereotypes about beauty-body vs. talent-strength is associated with a 0.50SD increase in the gender gap in *ThinkTooFat* (N = 34, see Table 4 and Table S3Bi in S1 File).

Besides, we observe that the relation is mainly due to girls and that across countries, higher levels of gender talent stereotypes are significantly associated with greater levels of body dissatisfaction among girls but not among boys (Table S3Bi in S1 File). See Fig 2 for an illustration.

For certain measures of stereotypes, the association between gender differences in body dissatisfaction and gender stereotypes is stronger than that between these gender differences and a country’s level of development (Table S3Bii in S1 File). For instance, the correlation between the gender gap in *ThinkTooFat* and gender stereotypes about talent (*r* = 0.73) is stronger than its correlation with HDI (*r* = 0.6) or GDP (*r* = 0.53). Besides, when regressing the gender gap in *ThinkTooFat* on both gender talent stereotypes and HDI, the coefficient of HDI is no more significant, while the coefficient of gender stereotypes remains significant (Table 4 and Table S3Bii in S1 File). A similar pattern is observed when replacing HDI by the log of GDP (Table 4 and Table S3Bii in S1 File), or when replacing the gender gap in body dissatisfaction by girls’ level of body dissatisfaction (Table S3Biii in S1 File). For stereotypes about body/beauty, we observe that the relation with the gender gap in *ThinkTooFat* is robust to controlling for HDI or GDP, but the relation with these indicators of country development remains significant (Table 4 and Table S3Biii in S1 File). This may be due to the noisy measure of stereotypes obtained through word embeddings. This simple regression analysis suggests that gender stereotypes may at least partly explain the relation between a country’s level of development or wealth and gender gaps in body dissatisfaction (the GEP in body dissatisfaction) or girls’ levels of body dissatisfaction.

## Discussion

After summarizing our main results, we first discuss and interpret them, particularly in relation to existing sociocultural theories such as Objectification Theory (OT) and the Tripartite Influence Model (TIM). We then analyze their implications, and, finally, highlight the limitations of our approach.

### Summary of the results

Relying on two large-scale representative surveys, we have shown that adolescent boys and girls differ significantly in terms of body dissatisfaction. Girls consistently exhibit higher levels of body dissatisfaction than boys (R1), regardless of BMI, of socioeconomic background, age, or country. Body dissatisfaction is significantly related to lower life satisfaction, self-esteem and well-being, and the relation is stronger for girls than for boys, which can be interpreted as a higher centrality of body dissatisfaction in girls’ lives than boys’ (R2). We found marked heterogeneity in effects sizes across subgroups, with the gender gap in body dissatisfaction being stronger among participants with a higher BMI, older age (15–16 years old), higher academic performance, and higher socioeconomic status (R3).

We have also shown considerable variation in the gender gap across countries, primarily driven by differences in girls’ body dissatisfaction between countries (R4). Countries with larger gender gaps in body dissatisfaction also show greater gender gaps in life satisfaction, eating disorders and depression (R5). Additionally, body dissatisfaction satisfies the Gender Equality Paradox; the gender gap in body dissatisfaction is stronger in more developed and gender equal countries, mainly due to the higher levels of body dissatisfaction among girls in these countries (R6).

Stereotypes essentializing boys and girls, like those associating women with their body and beauty rather than with talent or career appear stronger in economically developed countries (R7). Such stereotypes are related to gender gaps in body dissatisfaction at the country-level, these relations being primarily driven by variations in girls’ body dissatisfaction (R8). Gender stereotypes may (partly) account for the gender equality paradox in body dissatisfaction, and for the association between girls’ body dissatisfaction and a country’s development (R9); specifically, stronger appearance-related stereotypes in more developed countries may help explain both girls’ higher levels of body dissatisfaction and larger gender gaps.

### Interpretation

Objectification theory (OT, [10]) posits that the societal view of the female body as an object to be looked at and evaluated according to appearance standards influences women’s feelings about their bodies [54]. Men as well as women are inclined to evaluate women primarily in terms of their appearance, rather than their accomplishments, whereas they do not evaluate men in this way [10]. As a result, looks dominate our judgment of the general worth of women, as if women were represented by their body [55]. Through the experience of objectification, women come to view themselves as objects to be appreciated by others, they learn to internalize an observer’s view of their own body and to evaluate it relative to prevailing social ideals (self-objectification) leading to body dissatisfaction. Similar insights can be traced back to Beauvoir [56]. The tripartite influence model (TIM, [11]) complements this perspective by emphasizing three sociocultural sources of pressure: media/cultural influences, parental influences, and peer influences, which shape body dissatisfaction primarily through internalization of appearance ideals and social comparison. In contexts where the pressures are stronger, TIM predicts higher body dissatisfaction. More broadly, these perspectives align with foundational work on physical appearance and gender that integrates sociobiological and sociocultural viewpoints [57]. OT and TIM provide a good framework to understand our results.

First our result (R1) that girls experience more body dissatisfaction than boys is fully in line with abundant previous empirical results, obtained on less diverse samples or in meta-analyses [17,18,20,21] as well as with OT, according to which self-objectification primarily affects girls and women [58,59]. Our result (R2), which shows that body dissatisfaction is more related to life satisfaction, well-being and self-esteem in general in girls than in boys, is also in line with previous literature [1,3,9,60–63] and with OT, according to which girls and women are represented by their body, making satisfaction with their body and appearance central to their identity and self-worth.

As sociocultural theories, OT and TIM suggest that girls’ body dissatisfaction is shaped by the societal view on female bodies, which can explain why girls’ level of body dissatisfaction varies across countries and cultures and more than that of boys (R4). Consequently, this also helps to explain the significant variation in the gender gap in body dissatisfaction across countries. The fact (R5) that across countries gender gaps in body dissatisfaction are related to gender gaps in life satisfaction, eating disorders and depression, this relation being driven by girls, are fully in line with theoretical results from OT and empirical results relating at the individual level body dissatisfaction to life dissatisfaction, eating disorders and depression, especially in girls.

According to OT and TIM, cultural environment and societal gender norms influence women’s self-objectification, and countries with stronger gender stereotypes associating women with their appearance rather than with their abilities should have greater gender gaps in body dissatisfaction, which is our result (R8). Moreover, since these stereotypes tend to be stronger in more developed countries (as shown in R7), these stereotypes could lead to greater gender differences in more developed countries, which is our results (R6) and (R9).

How can we interpret that stereotypes associating women with beauty are stronger in more developed countries (R7)? This finding aligns with previous literature on gender-essentializing stereotypes, such as those associating boys with career, math or talent, and girls with family or the arts, which tend to be stronger in more developed countries [39,40,47,52]. One explanation given in this literature is that as women increasingly participate in economic and social life, they often do so in traditionally female-typed roles, thereby reinforcing these stereotypes. This dynamic sustains a clear distinction between the sexes, even as gender equality advances in other domains.

Another related argument concerns the emphasis on personal identity in wealthy and individualistic societies. These societies often highlight the importance of individual traits, preferences, and the concept of a “true self.” As noted in [38], these notions of self are heavily influenced by socially constructed ideas (see also [64]), including traditional gender stereotypes, which can lead to the reinforcement of such stereotypes in individualistic contexts.

To these explanations that are valid for gender-essentializing stereotypes in general can be added explanations that are specific to the stereotypes associating physical appearance to women, such as the fact that media images, the fashion industry and advertising, that are more prevalent in more developed countries, may increase the objectification and sexualization of female bodies. Murnen and Don [9] observe an increasingly sexualized image of women in the media and an emphasis on women’s appearance in the US, and suggest that the focus on appearance may have increased as a type of backlash (see also [19] for similar observations).

The results (R3) remain to be interpreted. The relation between gender gaps in body dissatisfaction and age (i.e., higher at 15 years old than at 11 years old) has already been found in previous literature on smaller samples or in meta-analyses [41,65–67]. Possible explanations include the fact that adolescence constitutes a stage of strong social pressures among peers, to adjust to gender and beauty norms (see also [64]).

Other mechanisms, consistent with OT and TIM, may help explain the association between high academic performance and high socio-economic status with greater gender gaps in body dissatisfaction (R3). One possible explanation is that high-performing girls, who enter traditionally male-dominated domains, may face identity costs. Similar to how women in male-dominated jobs are viewed as “unfeminine” [68], high-performing girls might be seen as “not true girls” or “not feminine”. Their appearance might be scrutinized more harshly, or they may focus more on their looks to compensate and keep their feminine identity, leading to increased body dissatisfaction. Another possible explanation is that high-performing girls may strive for “perfection” in all areas, including the correspondence to the feminine ideal, leading to greater body dissatisfaction. Regarding high socioeconomic status, gender identity concerns may be more pronounced. As Goudeau and Cimpian [69] note, self-construals are more prevalent in upper-class contexts. Additionally, the value placed on one’s body and body ideals may vary by socioeconomic status; for instance, women from lower socioeconomic backgrounds may prioritize physical strength, making them less vulnerable to societal beauty ideals [9]. Swami [70] observes that women in low SES contexts tend to experience significantly lower body dissatisfaction compared to their counterparts in high-SES contexts (see also [71,72]), and refers to the thin ideal that seems more prevalent in high SES contexts. Moreover, wealthier women may have more resources and opportunities to invest in managing and improving their bodies, leading to greater body concerns (this argument also applies to explaining higher female body dissatisfaction in wealthier countries). It is also possible that the tendency to evaluate girls based on their appearance is more prevalent in higher socioeconomic groups.

### Theoretical and practical implications

#### Theoretical implications.

Our analysis first offers strong support for sociocultural models of body dissatisfaction. As detailed above, our conclusions are fully in line with Objectification Theory and the Tripartite Influence Model. In particular, we have highlighted how effect sizes vary depending on context, including an individual’s socio-economic status, age and the social norms of their country.

Besides, our analysis provides new insights into the gender equality paradox. It first shows that body dissatisfaction satisfies the GEP. This result is interesting in its own right, but it also sheds light on the GEP in well-being or self-esteem. It suggests that body dissatisfaction may be one of the paths through which more developed countries exhibit greater gender gaps in life satisfaction and self-esteem. Importantly, in line with [40], our analysis suggests that stereotypes are likely part of the explanation of the Gender Equality Paradox (in body dissatisfaction). Indeed, we have shown that stereotypes associating women with physical appearance are stronger in more developed countries and that these stereotypes may at least partly account for the relation between a country’s level of development and gender gaps in body dissatisfaction.

Our approach also supports the growing literature underlining the multidimensional nature of gender equality [38,73–76]. Indeed, we show that in more developed and gender equal countries, more gender egalitarian values and practices can coexist with stronger stereotypes about women’s appearance and stronger imbalances in body dissatisfaction. In these countries, men and women are at the same time considered more equal and more different.

#### Practical implications.

Our analysis shows that body dissatisfaction disproportionately affects girls compared to boys. At the country level, we have confirmed a link between gender differences in body dissatisfaction and gender differences in eating disorders and other mental health issues. At the individual level, we have confirmed the strong association between body dissatisfaction and lower life satisfaction, well-being, and self-efficacy, with these associations being particularly pronounced for girls. Higher concerns about physical appearance drain cognitive and emotional resources, leaving women with less mental energy for other pursuits and leading to feelings of powerlessness and anxiety, ultimately limiting their engagement in personal achievements, relationships, and professional growth. Besides, while women are often judged on their appearance, physical attractiveness is associated with lower perceptions of competence, creating an inherent disadvantage. Taken together, these elements suggest that a focus on their appearance and body dissatisfaction represent a significant disadvantage for girls, constituting a form of gender inequality.

Our findings on the Gender Equality Paradox in body dissatisfaction, along with the stronger stereotypes about women’s physical appearance in more developed and gender-equal countries, suggest that gender gaps in body dissatisfaction are unlikely to diminish on their own as societies develop. Directly addressing stereotypes is difficult. An initial step could be to remain vigilant in rejecting the association of women with their appearance in the media. Another approach is to raise awareness among both men and women about the gender inequality tied to society’s treatment of women’s body and the range of adverse psychological effects that it generates. Additional approaches include featuring a wider variety of body shapes in the media, promoting role models who are valued for other qualities than their appearance, and more generally, to stop essentializing men and women.

### Limitations

There are limits to our work.

First, while our samples are large, representative and span a substantial number of countries, they have certain limitations. They are restricted to adolescents aged 11–16, excluding adults, which limits the generalizability of our findings to older age groups. Indeed, although adolescence is a particularly relevant period for studying body dissatisfaction due to the emergence of body-related health issues at that age, gender differences in body dissatisfaction may vary significantly with age. Besides, age-specific factors, such as social desirability bias—which is particularly prevalent among adolescents—may lead respondents to provide answers aligned with societal expectations, potentially influencing the observed gender differences. Additionally, while the HBSC dataset covers a relatively broad range of 40 countries, the PISA data is limited to just 9 countries. Furthermore, our samples do not allow for the consideration of individuals not conforming to the binary gender classification, nor for the exploration of intersectionality issues or of variations in body dissatisfaction experiences among diverse minority groups.

Second, our measures of body dissatisfaction are not exempt from criticism. In the HBSC dataset, we focus solely on issues related to fatness, excluding concerns around thinness and muscularity, which tend to be more relevant for males (see [9]). While these concerns are less likely to be related to eating disorder and depression, they may be linked to other negative outcomes, that we do not address. However, the PISA data allow us to show the consistency of the conclusions across different conceptualizations of body dissatisfaction, such as body weight, general body dislike, and appearance (albeit for a more limited set of countries). In both datasets, we lack a measure of *worry* or preoccupation with one’s body, which could provide valuable insights; indeed, individuals may report satisfaction with their bodies, but only at the cost of significant time, worry [77] or even surgical interventions [78].

Additionally, our measures of body dissatisfaction rely on self-reported data, which raises the possibility that women may report actual distress, particularly dissatisfaction with their bodies, more readily than men. This could be influenced by social gender norms around stoicism, potentially contributing to the observed gender gap in body dissatisfaction (and similar gender gaps in life satisfaction or well-being reported in the literature). However, this explanation does not seem able to account for the variation of the gender gap across factors such as BMI, academic performance, socioeconomic background or country development, as it is unclear why women, but not men, would selectively report more their distress in these contexts. Furthermore, this explanation fails to account for stronger gender stereotypes about physical appearance in more developed countries. Relatedly, some measures used in our analyses, such as BMI, rely on self-reported height and weight, which can be affected by gendered reporting or perception biases; for instance, girls may be more likely to overestimate their weight status, whereas boys may be more likely to overestimate their height. Such biases could (modestly) influence BMI-based comparisons.

Future research could use qualitative approaches (e.g., interviews, focus groups, or ethnographic work) to explore the lived experiences underlying these survey patterns. Such work could clarify how adolescents interpret body-related questions, how appearance norms are negotiated in everyday settings (school, peers, social media), and whether the meanings of “body dissatisfaction” differ across gender and cultural contexts. This would provide a richer understanding of the mechanisms suggested by our cross-country findings and help refine measurement in future large-scale surveys.

Third, measuring stereotypes, especially across different countries, is inherently challenging and our measures of gender stereotypes, whether directly or indirectly related to the association of women with appearance, are not without limitations. We drew on existing measures of stereotypes from previous studies [39,47,53]. To more specifically capture the association of women with attributes like body and beauty, we used word embeddings, a method that has proven effective in detecting biased associations and cultural beliefs within societies. However, word embeddings can introduce noise, particularly in cross-country analyses. Factors such as corpus selection, choice of stimuli, and translation may all influence the measurement of biased associations. In our view, the most compelling evidence is the convergence of findings across multiple stereotype measures, each based on distinct constructs, elicitation methods, and/or surveys.

## Supporting information

S1 File Supporting information for *Gender differences in Body Dissatisfaction: a large-scale investigation among adolescents using two international surveys.* (DOCX)
