# Supplementary material for: Gender differences in body dissatisfaction: A large-scale investigation among adolescents using two international surveys
Source: PLoS One. 2026 Mar 6;21(3):e0330766. doi: 10.1371/journal.pone.0330766 (PMC12965547; doi:10.1371/journal.pone.0330766)
Supplement: S1 File — (DOCX) [file pone.0330766.s001.docx]

Supporting Information for

**Gender differences in Body Dissatisfaction: a large-scale investigation among adolescents using two international surveys**

Clotilde Napp^a,b,*^

^a^ CNRS, UMR7088, 75016 Paris, France

^b^ Université Paris-Dauphine, PSL Research University, Paris, France

^*^Corresponding author: Email: [clotilde.napp@dauphine.psl.eu](mailto:xxxxx@xxxx.xxx)

**This PDF file includes:**

Supplementary Text: Appendix A, Appendix B

Tables S1 to S3

[Supplementary Text 2](#_Toc216352317)

[Appendix A: Data 2](#_Toc216352318)

[General description of PISA2018 and HBSC2018 2](#_Toc216352319)

[Variables of interest in PISA2018 2](#_Toc216352320)

[Variables of interest in HBSC2018 4](#_Toc216352321)

[Data on country-level gender stereotypes 5](#_Toc216352322)

[Other data sources 6](#_Toc216352323)

[Appendix B: Methods 7](#_Toc216352324)

[Weights and representativeness 7](#_Toc216352325)

[Standardized variables 7](#_Toc216352326)

[Regressions 8](#_Toc216352327)

[Appendix C: Additional Tables 9](#_Toc216352328)

Supplementary Text

# Appendix A: Data

## General description of PISA2018 and HBSC2018

The **Programme for International Student Assessment (PISA)** is an every-three-year international survey of 15-year-old students aimed at determining their knowledge and skills in different domains. Students' abilities are assessed in the three curricular domains: mathematics, reading, and science. Students also answer a background questionnaire, seeking information about the students themselves, their homes, and their school and learning experiences. We analyse data from the PISA 2018 survey.

For the first time, PISA2018 distributed an optional well-being questionnaire about students’ satisfaction with different aspects of their lives, like their health, their life at school, the way they look. Questions about students' body dissatisfaction are included in the well-being questionnaire. PISA2018 includes more than 70 countries, but only 9 countries distributed the well-being questionnaire. The sample of teenagers responding to the well-being questionnaire in 2018 consists of 71,769 observations in 9 countries with complete information on body satisfaction with OECD countries like Spain (25,185 obs), Ireland (4,865 obs), Mexico (5,612 obs) and non-OECD countries like Bulgaria (3,464 obs), Georgia (3,844 obs), Hong-Kong (4,693 obs), Panama (4,219 obs), Serbia (4,288 obs), United Arab Emirates (15,599 obs). The national samples are representative of all 15-year-old students in the country. PISA also includes in the well-being questionnaire questions about the student's height and weight (WB151q01ha and WB152q01ha), which permits to determine individual Body Mass Index (BMI), as informed by the students.

Moreover, PISA2018 also includes in the background questionnaire information about students' socioeconomic status, but also their life satisfaction, negative and positive feelings, meaning in life, self-efficacy, which permits to analyze their relationship with body dissatisfaction.

The **Health Behaviour in School-Aged Children (HBSC)** survey is conducted by the World Health Organization (WHO) and its regional offices. The HBSC survey is a collaborative cross-national study that has been run every four years since 1983. It includes 11-, 13- and 15-year-olds school children in representative samples of schools in each of the participating countries and collects data about their health, well-being, and social environments.

We analyze data from the HBSC2018 survey. It includes information on body satisfaction for a total of 229,327 children in 40 different countries (see list in Table S2A). HBSC2018 survey also includes information about body mass index, family affluence and about life satisfaction, which permits to analyze their relationship with body dissatisfaction.

## Variables of interest in PISA2018

**Body dissatisfaction and other body-related items.** Several questions in the well-being questionnaire deal with students' body, look and appearance. Questions WB153 are about a student’s body image. Students are asked, thinking about themselves, how much they agree with each of the following statements: 'I like my look just the way it is' (WB153q01ha), 'I consider myself to be attractive' (WB153q02ha), 'I am not concerned about my weight' (WB153q03ha), 'I like my body' (WB153q04ha), 'I like the way my clothes fit me' (WB153q05ha). Answers are given on a four-point Likert scale with response categories ranging from “Strongly disagree”, “Disagree”, “Agree”, to “Strongly agree”. An additional response option “I don’t have an opinion” was treated as missing. PISA constructs the index of body image relying on all 5 questions WB153.

An additional question WB155q02ha asks students how satisfied they are with the way they look. Answers range from 1 (not at all satisfied) to 4 (totally satisfied).

For all these body and appearance items and index, we reverse the scale so that higher scores correspond to higher dissatisfaction with one's body.

We take as *our main measure of body dissatisfaction (BD),* the reverse-coded PISA index, relying on all 5 questions WB153.

For the sake of robustness and reliability, we consider four alternative measures of body dissatisfaction: three measures of specific aspects of body dissatisfaction, namely the concerns about one's weight, the dislike of one's body in general, and the dissatisfaction about one's look or appearance, as well as an average of these measures:

- the *body weight concern or dissatisfaction*, measured by the reverse-coded item 'I am not concerned about my weight' (WB153q03ha),
- the *general body dislike or dissatisfaction*, measured by the reverse-coded item 'I like my body' (WB153q04ha).
- the *body look dissatisfaction* measured by the reverse coded item 'how satisfied are you about the way you look' (WB155q02ha)
- the weight and appearance dissatisfaction measured by the equally weighted average of the (reverse-coded) items 'I am not concerned about my weight' (WB153q03ha), 'I like my body' (WB153q04) and 'how satisfied are you about the way you look' (WB155q02).

In order to measure, at the country level, the percentage of boys and girls who are concerned about their body weight, or don't like their body and the gender gap, we introduce the binary variable equal to one when the student answers either “Strongly disagree” or “Disagree” to question WB153q03ha or WB153q04ha.

**Students' body mass index, socioeconomic background and academic performance**

- PISA includes in the well-being questionnaire questions about the student's height and weight (WB151q01ha and WB152q01ha), which permits to determine individual *Body Mass Index* (BMI), as reported by the students. We recall that Body mass index (BMI) is a person's weight in kilograms divided by the square of height in meters
- In PISA, a student’s socio-economic background is estimated by the PISA index of *economic, social and cultural status* (ESCS), which is based on information about parental education, highest parental occupation, and home possessions including books in the home.
- We use individual-level PISA scores in math, reading and science to measure *students’ performance.* These scores are on a 0-1000 scale. They have been scaled during the first PISA survey in 2000 to have a mean of 500 and a standard deviation of 100. We will also consider general performance, measured as an equally weighted average of the three scores in math, reading and science. Performances in math, reading and science are highly correlated and we sometimes use science performance instead of general performance because science scores are available for all students, whereas reading scores, hence general scores, are not available for Spain.

**Students' life satisfaction, negative feelings and sense of self-efficacy.** The standard students' questionnaire in PISA2018 includes questions about students' life satisfaction, feelings and self-efficacy.

- In question ST016q01na, students are asked to rate their *life satisfaction* ('Overall, how satisfied are you with your life as a whole these days?') on a scale from 0 (not at all satisfied) to 10 (completely satisfied).
- In questions ST188 students are asked to report the extent to which they agree (“strongly disagree”, “disagree, “agree”, “strongly agree”) with the following statements about themselves: “I usually manage one way or another”; “I feel proud that I have accomplished things”; “I feel that I can handle many things at a time”; “My belief in myself gets me through hard times”; and “When I’m in a difficult situation, I can usually find my way out of it”. These statements were combined to create the index of *self-efficacy* (RESILIENCE). Positive values in this index mean that the student reported higher self-efficacy than did the average student across OECD countries.
- In questions ST186, students are asked to report about their positive and negative feelings and in particular how frequently (“never”, “rarely”, “sometimes”, “always”) they *feel miserable*.
- In questions ST185, students are asked to report the extent to which they agree (“strongly agree”, “agree”, “disagree”, “strongly disagree”) with the following statements: “My life has clear meaning or purpose”; “I have discovered a satisfactory meaning in life”; and “I have a clear sense of what gives meaning to my life”. These statements were combined to form the index of *meaning in life* (EUDMO). Positive values in the index indicate greater meaning in life than the average student across OECD countries.
- In questions ST186, students are asked to report how frequently (“never”, “rarely”, “sometimes”, “always”) they feel happy, joyful, cheerful. These positive feelings were combined to create an index of *subjective well-being for positive feelings* (SWBP). Positive values in this index mean that the student reported more positive feelings than the average student across OECD countries.

**Other variables of interest**

- Individual controls include grade repetition, a measure of attitude towards school, namely how much students think that they belong to school, the level of education of the student’s parents, measured both in years and kind of diploma obtained, a measure of home educational resources.

## Variables of interest in HBSC2018

**Body dissatisfaction.** HBSC2018 includes a single question related to body image and appearance: "Do you think your body is a) much too thin, b) too thin, c) about right, d) too fat or e) much too fat?" We take as our main measure of body dissatisfaction the *ThinkTooFat* measure, which is equal to 1 if participants answered "too fat" (d), to 2 if participants answered "much too fat" (e) and to 0 otherwise.

**Life dissatisfaction** *Life satisfaction* is measured in HBSC survey using the Cantril Ladder (Cantril 1965), where teenagers were asked to pick a number from 0 (‘worst possible life’) to 10 (‘best possible life’) presented as steps on a ladder to indicate their general level of life satisfaction: "Here is a picture of a ladder. The top of the ladder ‘10’ is the best possible life for you and the bottom ‘0’ is the worst possible life for you. In general, where on the ladder do you feel that you stand at the moment? Tick the box next to the number that best describes where you stand. This measure is supposed to reflect not just current emotions but to be reliable for evaluating respondents' lives amidst the wavering experiences of adolescence.

**Other variables of interest: Body mass index, age, family affluence**

HBSC2018 survey also provides information about

- *Body Mass Index* (see definition above). The variable (MBMI) provides the (self-reported) body mass index of the participant.
- *Age category*: The variable (agecat) provides the age category of the participant: 1. for 11year-olds., 2. for 13year-olds. and 3. for 15year-olds.
- *Family affluence*: HBSC provides information about the socioeconomic status of school-aged children and adolescents in item (IRFAS), using the Family Affluence Scale (FAS). We consider three categories: low (IRFAS below 6), medium (IRFAS between 6 and 10) and high (IRFAS above 10).

## Data on country-level gender stereotypes

**Gender stereotypes about brilliance.** Cross-country measures of stereotypes about boys' brilliance or talent are provided in (1). They are based on national differences between girls and boys in their perceived lack of talent in PISA2018. More precisely, the measure of the prevalence of gender stereotypes about talent consists of the gender gap in students' agreement with the following assertion about themselves: *'When I am failing, I am afraid that I might not have enough talent'*, standardized by country, and controlling for students’ performance in math, reading, and science, which are also standardized by country. See (1) for more details about the method.

*Source*: (1)

**Gender stereotypes about career and family**

Country-level measures of implicit gender stereotypes regarding career and family, as well as explicit gender stereotypes about career, are provided in (2). It should be noted that, for implicit stereotypes, career and family stereotypes cannot be separated. In this study, we focus on implicit stereotypes of male participants and explicit stereotypes of female participants, as (2) demonstrates that these stereotypes are most closely associated with real-world outcomes

- Implicit measures provided in (2) rely on the implicit association test (IAT) and are based on data from Project Implicit (3) (http://implicit.harvard.edi/implicit/) collected during the years 2005-2020. In Project Implicit, participants are asked to categorize typical men (Ben, Paul, Daniel, John, Jeffrey) or women names (Rebecca, Michelle, Emily, Julia, Anna), as well as words related to career (Career, Corporation, Salary, Office, Professional, Management, Business) and family (Wedding, Marriage, Parents, Relatives, Family, Home, Children). In one condition (“stereotypical condition”), participants categorize men and career words with the *e* key, and women and family words with the *i* key. In the other condition (“counter-stereotypical condition”), participants categorize women and career words with the *e* key, and men and family words with the *i* key. Implicit gender stereotypes about career and family are measured at the individual level by the difference in response time between the two IAT conditions, which likely reflects the stereotypical association in memory of men with career and of women with family. We consider the implicit gender stereotypes of male participants only, averaged by country. See (2) for more details about the method.
- Explicit measures of the association of career with men provided in (2) also rely on data from Project Implicit (3) (http://implicit.harvard.edi/implicit/) collected during the years 2005-2020. Participants are explicitly asked how strongly they associate career with men or women. Answers are given on a seven-point Likert scale, providing a measure of the explicit stereotype associating career with men relative to women. We consider the answers of female participants only, averaged by country.

*Source*: (2)

**Association of *sexy* with females vs. males.** In their multination study about gender stereotypes (4, 5), Williams and Best report for 300 psychological traits, described by an adjective from the Adjective Check List, a stereotypical^[[1]](#footnote-1)^ masculinity/femininity score. This score represents the extent to which the trait is stereotypically associated with men or women in each country in their sample (N=26). We consider the national masculinity/femininity scores for the adjective *sexy*. We consider the opposite of the masculinity/femininity score, so that a higher value corresponds to a higher stereotypical association of the adjective *sexy* to women.

*Source*: (4, 5)

**Gender stereotypes about Beauty/Body vs. Talent/Strength relying on large text corpora.** We introduce country-level measures of gender stereotypes about beauty or body vs. talent or strength. Our approach is similar to the one in (6) and exactly the same as in (7), replacing gender stereotypes about career-family by gender stereotypes about beauty-talent. We rely on publicly available word embeddings pretrained on text corpora from Wikipedia (8) using the fastText algorithm (a variant of word2vec (9)). We consider linguistic stereotypical gender associations about beauty-talent, beauty-strength, beauty/body-talent/strength. For each stereotype, we choose stimuli, *i.e.,* sets of words representing the categories men and women as well as sets of words representing the attributes (beauty-talent, beauty-strength, beauty/body-talent/strength). We choose the simplest stimuli, i.e., ["man"] for the men category, ["woman"] for the women category, ["beauty"] for the beauty category, ["talent"] for the talent category, ["strength"] for the strength category, and finally, [["beauty", "body"] for the beauty/body category and ["talent", "strength"] for the talent/strength category.

We translate these stimuli for all other languages using ChatGPT. As in (6), we rely on the Word Embedding Association Test (WEAT) to obtain our measures of gender stereotype. The Word Embedding Association Test (WEAT) tweaks the Implicit Association Test for word embeddings and permits to measure the extent to which the word "woman" is more similar than the word "man" to the word "beauty" versus the word "talent", to the word "beauty" versus the word "strength", and to the words "beauty" or "body" versus the words "talent" or "strength". See details about the method in (7).

## Other data sources

**Data on measures of country development, wealth, gender equality and individualism**

*Human Development Index (HDI)*

The Human Development Index (HDI) is a composite statistic of life expectancy, education, and per capita income indicators. A country scores a higher HDI when life expectancy, education level and per capita income is higher. Values have been taken for year 2018.

*Source: http://hdr.undp.org/en/content/2019-human-development-index-ranking*

*Gross Domestic Product (GDP)*

GDP per capita is gross domestic product divided by midyear population. GDP is the sum of gross value added by all resident producers in the economy plus any product taxes and minus any subsidies not included in the value of the products. It is calculated without making deductions for depreciation of fabricated assets or for depletion and degradation of natural resources.

*Source:https://data.worldbank.org/indicator/NY.GDP.PCAP.PP.KD?end=2019&most_recent_year_desc=true&start=2018*

*Gender Gap Index (GGI)*

The Gender Gap Index, from the World Economic Forum, synthesizes the position of women in any given country by taking into account economic opportunities, economic participation, educational attainment, political achievements, and health and well-being. Larger values point to a better position of women in society.

*Source: http://www3.weforum.org/docs/WEF_GGGR_2018.pdf*

*Individualism*

Individualism is based on Hofstede’s cultural dimensions*.* It captures the degree to which a society is individualistic (as opposed to collectivist), *i.e.* the extent to which individuals are integrated into groups, and how loose are social links.

*Source:* *https://www.hofstede-insights.com/product/compare-countries/*

**Data on eating disorders and depression**

Mental health disorders remain widely under-reported. This is true across all countries, but particularly at lower incomes where data is scarcer, and there is less attention and treatment for mental health disorders. Data should be taken as estimates of mental health disorder prevalence. We hope that it does not affect too much estimates of the gender gap in mental health disorders. Most of the estimates are produced by the Institute for Health Metrics and Evaluation (IHME) and reported in their flagship Global Burden of Disease study.

*Eating disorders*

Eating disorders incorporates a spectrum of disordered eating behaviors. We present data only for the disorders of clinically diagnosed anorexia and bulimia nervosa. It is however recognized that a large share of eating disorders falls outwith the definition of either anorexia or bulimia nervosa. The prevalence of clinically diagnosed anorexia and bulimia nervosa ranges from 0.1 to 1% by country. Values have been taken for year 2017 and we have considered all countries participating in PISA2018.

*Source: https://ourworldindata.org/mental-health#eating-disorders*

*Depression or depressive disorders*

Depressive disorders occur with varying severity. We include all forms of depressive disorders from mild, to persistent depression (dysthymia) and major depressive disorder (severe). The share of population with depression ranges mostly between 2% and 6% around the world today. Values have been taken for year 2017 and we have considered all countries participating in PISA2018.

*Source: https://ourworldindata.org/mental-health#depression*

# Appendix B: Methods

## Weights and representativeness

PISA and HBSC provide weights to make surveyed students representative of the 15-year-old students (PISA) or 11-15 y. o. teenagers (HBSC) in the surveyed countries. We use these weights in all country-specific and whole sample analyses, so that all the results we provide are not subject to sample selection and are consistent estimates of the underlying parameters at the country level.

Moreover, we use senate weights (i.e., participants' weights normalized to sum to one in each country), so that each country has an equal influence in the regressions and other analyses instead of contributing according to its total population.

## Standardized variables

In both PISA and HBSC surveys, when considering gender gaps in body dissatisfaction, to get results that can be interpreted both on our whole sample and country-by-country, we normalize the measures of body dissatisfaction so that their weighted mean is zero in each country of the sample, while their weighted standard deviation is one in each country of the sample. Gender gaps in body dissatisfaction are then directly expressed as a fraction of its standard deviation and, as such, directly comparable across countries. This standardization implies that our measures of body dissatisfaction have the same mean and standard deviation in each country, so that our main results are not driven by cross-country differences in the variable.

For the same reason, we standardize similarly (“at the country level”) measures of well-being, life satisfaction and self-efficacy when considering gender differences.

Items are not standardized when comparing boys' and girls' levels across countries. We always consider for instance non-standardized body dissatisfaction items when we analyze how boys' and girls' body dissatisfaction evolve with countries' level of development or gender equality.

Finally, dummy variables for which gender gaps can be simply expressed as a percentage points’ difference in the rate of positive answers are never standardized.

## Regressions

Empirical analyses presented in the paper rely primarily on individual-level regressions that control for some observable individual characteristics as well as on country-level correlations and multivariate regressions with a few competing explanatory variables.

# Appendix C: Additional Tables

**Table S1A. Gender gaps in body dissatisfaction for various measures of body dissatisfaction**

|  | **GENDER**  **GAP** |
| --- | --- |
|  |  |
| **PISA SAMPLE** | **PISA2018** |
| ***Body dissatisfaction BD*** | 0.15*** |
| ***General Body Dislike*** | 0.20*** |
| ***Body Weight Concern*** | 0.20*** |
| ***Body Look Dissatisfaction*** | 0.17*** |
| ***Weight and Appearance Dissatisfaction Index*** | 0.25*** |
|  |  |
| **HBSC SAMPLE** | **HBSC2018** |
| ***ThinkTooFat*** | 0.19*** |

Notes: The table presents gender gaps in body dissatisfaction measures across two different samples: the PISA sample (N=71,769 observations, 9 countries) and the HBSC sample (N= 229,327 observations, 40 countries). Measures of Body dissatisfaction in the PISA sample rely on 6 different items in PISA survey and are defined in main text and in Appendix A. Our primary measure of body dissatisfaction (*BD*) is the (reverse-coded) body image index constructed by PISA, relying on students' agreement with the five following statements: 'I like my look just the way it is', 'I consider myself to be attractive', 'I am not concerned about my weight', 'I like my body', 'I like the way my clothes fit me'. We also consider a measure of the participant's self-reported dissatisfaction about one's look (*Body Look Dissatisfaction* relying on participants answer to the question “how satisfied are you about the way you look?”), a measure of the participant's concerns about one's weight (*Body weight Dissatisfaction*, relying on the participant's level of disagreement with the item ''I am not concerned about my weight'), a measure of the participant's dislike of one's body in general (*General Body dislike*, relying on the participant's level of disagreement with the item: 'I like my body'), as well as an average of these three measures (the *Weight and Appearance Dissatisfaction Index*). The measure *ThinkTooFat* of Body dissatisfaction in the HBSC sample relies on one question in HBSC survey about what the participants think of their body, being equal to 1 if participants answer that they think their body is "too fat", to 2 if participants answer "much too fat" and to 0 otherwise. For all measures, a positive gender gap represents a higher dissatisfaction among girls. The variables are standardized to have a weighted mean equal to 0 and a weighted standard deviation equal to 1 in each country. **** p<0.01, ** p<0.05, * p<0.1*

|  | BOY | GIRL | GAP |
| --- | --- | --- | --- |
|  | (%) | (%) |  |
| **PISA2018 Sample** |  |  |  |
| **Percent concerned with weight** | 37,45% | 48,17% | 10,7*** |
| **Percent who don't like their body** | 25,86% | 35,05% | 9,2*** |
|  |  |  |  |
| **HBSC2018 Sample** |  |  |  |
| **Percent who think they are 'too fat' or 'much too fat'** | 20,6% | 28,65% | 8,1*** |
| **Percent who think they are 'much too fat'** | 2,4% | 4,3% | 1,9*** |

Notes: The table presents the percent of boys and of girls who are dissatisfied with their body as well as the gender gap for various measures of body dissatisfaction across two different samples. For the PISA sample (N=71,769 observations), we consider the percentage of boys and girls who answer that they 'strongly disagree' or 'disagree 'with the statement 'I like my body' as well as the percentage of boys and girls who answer that they 'strongly disagree' or 'disagree 'with the statement 'I am not concerned about my weight'. For the HBSC sample (N= 229,327 observations), we consider the percentage of boys and girls who answer that they think their body is 'too fat' or 'much too fat' as well as the percentage of boys and girls who answer that they think their body is 'much too fat'. **** p<0.01, ** p<0.05, * p<0.1*

| **Table S1B. Robustness of gender gaps in body dissatisfaction to various sets of control variables**  **PISA SAMPLE** | | | | | | |  |
| --- | --- | --- | --- | --- | --- | --- | --- |
|  | *Gender Gap in Body Dissatisfaction* | | | | | | |
|  | **(1)** | (2) | (3) | (4) | (5) | (6) | |
| **Averages** | | | | | | | |
| ***BD*** | 0,15*** | 0,18*** | 0,15*** | 0,15*** | 0,17*** | 0,13*** | |
| ***General Body Dislike*** | 0,20*** | 0,22*** | 0,20*** | 0,20*** | 0,22*** | 0,18*** | |
| ***Body Weight Concern*** | 0,20*** | 0,22*** | 0,20*** | 0,22*** | 0,23*** | 0,21*** | |
| ***Body Look Dissatisfaction*** | 0,17*** | 0,20*** | 0,17*** | 0,18*** | 0,20*** | 0,15*** | |
| ***Weight and Appearance Dissatisfaction Index*** | 0,25*** | 0,27*** | 0,24*** | 0,26*** | 0,28*** | 0,24*** | |
| **CONTROLS** | NONE | Individual  controls | Controls for Performance | Controls for Body Mass Index | Individual controls  +BMI  + Controls for Performance | +Controls for Well Being and self-esteem | |

Source: PISA 2018.

Notes: The table presents the gender gap in Body Dissatisfaction, without control (column 1) and with various controls (columns 2-6), relying on PISA sample. Body dissatisfaction is measured by five different variables described in detail in main text, in Appendix A and in the Notes of Table S1A. Variables are standardized to have a weighted mean equal to 0 and a weighted standard deviation equal to 1 in each country. Individual controls (column 2,5,6) include the level of education of the student’s parents, measured both in years and kind of diploma obtained, grade repetition, an index of economic, social and cultural status of the household, a measure of home educational resources, and a measure of attitude towards school, namely how much students think that they belong to school. Performance (column 3,5,6) is performance in science, standardized by country. BMI (column 4-6) is Body Mass Index and is described in Appendix A. Controls for well-being and self-esteem in the last column include students' levels of self-efficacy, of life satisfaction, of meaning in life, of negative feelings and an index of individual subjective well-being. These items are also described in Appendix A. **** p<0.01, ** p<0.05, * p<0.1*

**HBSC SAMPLE**

|  | *Gender Gap in Body Dissatisfaction* | |
| --- | --- | --- |
|  | (1) | (2) |
| **HBSC *ThinkTooFat*** | 0.19*** | 0.21*** |
| **CONTROLS** | NONE | BMI, age, family affluence, life satisfaction |
|  |  |  |

Source: HBSC 2018.

Notes: The table presents the gender gap in Body Dissatisfaction, without control (column 1) and with controls (column 2), relying on HBSC sample. The ThinkTooFat measure of body dissatisfaction is based on a single question in the HBSC survey about participants' perceptions of their body. It equals 1 if participants answer 'too fat,' 2 if they answer 'much too fat,' and 0 otherwise.A positive gender gap represents a higher dissatisfaction among girls. The variable is standardized to have a weighted mean equal to 0 and a weighted standard deviation equal to 1 in each country. Individual controls include the participant's age, Body Mass Index, level of family affluence, and level of life satisfaction. All measures and variables are described in Appendix A. **** p<0.01, ** p<0.05, * p<0.1*

**Table S1Ci. Gender gaps in body dissatisfaction among subgroups**

**PISA SAMPLE**

|  | ***BD*** | ***Body***  ***Dislike*** | ***Body***  ***Weight concern*** | ***Body***  ***Look Dissatisfaction*** | ***Weight and Appearance Dissatisfaction Index*** |
| --- | --- | --- | --- | --- | --- |
| **All** | 0.15*** | 0.20*** | 0.20*** | 0.17*** | 0.25^***^ |
|  |  |  |  |  |  |
| **BMI** |  |  |  |  |  |
| BMI <18.5 | -0.02 | 0.03^*^ | 0.10^***^ | 0.05^***^ | 0.07^***^ |
| Medium BMI | 0.22^***^ | 0.27^***^ | 0.27^***^ | 0.21^***^ | 0.33^***^ |
| BMI>25 | 0.16^***^ | 0.19^***^ | 0.19^***^ | 0.26^***^ | 0.27^***^ |
| **SOCIO-ECONOMIC STATUS** |  |  |  |  |  |
| Lower ESCS | 0.11^***^ | 0.18^***^ | 0.17^***^ | 0.13^***^ | 0.21^***^ |
| Higher ESCS | 0.19^***^ | 0.21^***^ | 0.22^***^ | 0.21^***^ | 0.28^***^ |
| **PERFORMANCE** |  |  |  |  |  |
| Lower science perf | 0.09^***^ | 0.16^***^ | 0.13^***^ | 0.10^***^ | 0.17^***^ |
| Higher science perf | 0.21^***^ | 0.23^***^ | 0.26^***^ | 0.23^***^ | 0.31^***^ |
| Lower overall perf | 0.06^***^ | 0.14^***^ | 0.12^***^ | 0.08^***^ | 0.15^***^ |
| Higher overall perf | 0.22^***^ | 0.23^***^ | 0.25^***^ | 0.23^***^ | 0.31^***^ |
| **ALL TOGETHER** |  |  |  |  |  |
| **Girl*ESCS** | 0.051*** | 0.041*** | 0.016* | 0.044*** | 0.050*** |
| **Girl*Performance** | 0.068*** | 0.038*** | 0.075*** | 0.058*** | 0.070*** |
| **Girl*BMI** | 0.080*** | 0.087*** | 0.058*** | 0.076*** | 0.085*** |
|  |  |  |  |  |  |

Source: PISA 2018.

Notes: The table presents the gender gap in Body Dissatisfaction across different groups in the PISA sample. Body Dissatisfaction is measured by the main indicator *BD*, as well as by alternative variables, all described in main text and in Appendix A (also see the Notes of Table S1A). For the five variables, a positive gender gap represents a higher dissatisfaction among girls. The variables are standardized to have a weighted mean equal to 0 and a weighted standard deviation equal to 1 in each country. The first row considers the whole sample. BMI denotes Body Mass Index, and we consider three groups according to the level of BMI. Socioeconomic status is measured by PISA index of economic, social and cultural status (ESCS), which is based on information about parental education, highest parental occupation, and home possessions including books in the home. Lower (resp. higher) ESCS is defined as the bottom (top) half of the level of ESCS in the country. Performance is performance in science as well as overall performance (average of math, reading and science performance), standardized by country. Lower (resp. higher) performance, well-being and self-efficacy corresponds to the bottom (top) half of the distribution of these outcomes in the country. The rows entitled 'All together' present the coefficient of the interaction of gender with ESCS, performance and BMI in the single regression of body dissatisfaction on a dummy for girl, ESCS, BMI, performance and their interaction with gender. **** p<0.01, ** p<0.05, * p<0.1*

**HBSC SAMPLE**

|  | ***ThinkTooFat*** |
| --- | --- |
| **All** | 0.19^***^ |
|  |  |
| **BMI** |  |
| BMI <18.5 | 0.12^***^ |
| Medium BMI | 0.28^***^ |
| BMI>25 | 0.34^***^ |
| **Age** |  |
| Low: 11 y.o. | 0.06^***^ |
| Medium: 13 y.o. | 0.23^***^ |
| High: 15 y.o. | 0.29^***^ |
| **IRFAS** |  |
| Low IRFAS | 0.16^***^ |
| Medium IRFAS | 0.19^***^ |
| High IRFAS | 0.22^***^ |
|  |  |
| **ALL TOGETHER** |  |
| **Girl*IRFAS** | 0.007* |
| **Girl*Age category** | 0.073*** |
| **Girl*BMI** | 0.081*** |
|  |  |
| **ALL TOGETHER 15y.o. only** |  |
| **Girl*IRFAS** | 0.0184** |
| **Girl*BMI** | 0.106*** |

Source: HBSC 2018.

Notes: The table presents the gender gap in Body Dissatisfaction across different groups in the HBSC sample. The measure *ThinkTooFat* of Body dissatisfaction relies on one question in HBSC survey about what the participants think of their body, being equal to 1 if participants answer "too fat", to 2 if participants answer "much too fat" and to 0 otherwise. A positive gender gap represents a higher dissatisfaction among girls. BMI denotes Body Mass Index, and we consider three groups according to the level of BMI. HBSC includes three age categories, and we consider them as subgroups. IRFAS represents the relative family affluence scale, and we consider three categories: low (IRFAS below 6), medium (IRFAS between 6 and 10) and high (IRFAS above 10). The rows entitled 'All together' present the coefficients of the interaction of gender with IRFAS (standardized by country), age and BMI (standardized by country) in the single regression of body dissatisfaction on a dummy for girl, age, IRFAS and BMI (both standardized by country) and their interaction with gender. The rows entitled 'All together 15-years-old only' present the same estimates but restricting the sample to 15-years-old only (and without the age coefficient in the regression). **** p<0.01, ** p<0.05, * p<0.1*

**Table S1Cii. Body Dissatisfaction among subgroups by gender**

**PISA SAMPLE**

|  | ***BD***  **GIRL** | ***BD***  **BOY** | ***Body***  ***Dislike***  **GIRL** | ***Body***  ***Dislike***  **BOY** | ***Weight***  **GIRL** | ***Weight***  **BOY** | ***Look***  **GIRL** | ***Look***  **BOY** | ***WADI***  **GIRL** | ***WADI***  **BOY** |
| --- | --- | --- | --- | --- | --- | --- | --- | --- | --- | --- |
| **All** | 2.024 | 1.882 | 2.226 | 2.062 | 2.437 | 2.256 | 2.145 | 2.019 | 2.278 | 2.116 |
|  |  |  |  |  |  |  |  |  |  |  |
| **BMI** |  |  |  |  |  |  |  |  |  |  |
| BMI<18.5 | 1.803 | 1.819 | 2.021 | 1.987 | 2.270 | 2.181 | 2.012 | 1.976 | 2.104 | 2.055 |
| Medium BMI | 1.974 | 1.769 | 2.206 | 1.984 | 2.446 | 2.197 | 2.103 | 1.949 | 2.259 | 2.044 |
| BMI>25 | 2.312 | 2.186 | 2.485 | 2.340 | 2.632 | 2.462 | 2.332 | 2.152 | 2.497 | 2.329 |
| **PERFORMANCE** |  |  |  |  |  |  |  |  |  |  |
| Lower science perf | 1.979 | 1.909 | 2.190 | 2.062 | 2.366 | 2.253 | 2.104 | 2.032 | 2.227 | 2.116 |
| Higher science perf | 2.063 | 1.856 | 2.258 | 2.062 | 2.499 | 2.259 | 2.181 | 2.006 | 2.322 | 2.116 |
| Lower overall perf | 1.977 | 1.932 | 2.184 | 2.072 | 2.355 | 2.255 | 2.094 | 2.042 | 2.218 | 2.123 |
| Higher overall perf | 2.056 | 1.842 | 2.254 | 2.054 | 2.491 | 2.257 | 2.179 | 2.000 | 2.317 | 2.111 |

Source: PISA 2018.

Notes: The table presents the level of Body Dissatisfaction by gender across different groups in the PISA sample. Body Dissatisfaction is measured by the main indicator *BD*, as well as by alternative variables (*General Body Dislike*, *Body Weight Concern*, *Body Look Dissatisfaction* and the *Weight and Appearance Dissatisfaction Index (WADI))*, all described in main text and in Appendix A (also see the Notes of Table S1A). Variables are not standardized. The first row considers the whole sample. BMI denotes Body Mass Index, and we consider three groups according to the level of BMI. Performance is performance in science, standardized by country. Lower (resp. higher) performance corresponds to the bottom (top) half of the distribution of performance in the country.

**HBSC SAMPLE**

|  | ***ThinkTooFat***  **GIRL** | ***ThinkTooFat***  **BOY** |
| --- | --- | --- |
| **All** | 0.350 | 0.245 |
|  |  |  |
| **BMI** |  |  |
| BMI <18.5 | 0.137 | 0.075 |
| Medium BMI | 0.426 | 0.274 |
| BMI>25 | 1.028 | 0.838 |
| **Age** |  |  |
| Low: 11 y.o. | 0.268 | 0.233 |
| Medium: 13 y.o. | 0.381 | 0.258 |
| High: 15 y.o. | 0.402 | 0.245 |
| **IRFAS** |  |  |
| Low IRFAS | 0.343 | 0.249 |
| High IRFAS | 0.361 | 0.239 |

Source: HBSC 2018.

Notes: The table presents the level of Body Dissatisfaction by gender across different groups in the HBSC sample. One question in HBSC survey asks what the participants think of their body, and the measure *ThinkTooFat* is equal to 1 if participants answer "too fat", to 2 if participants answer "much too fat" and to 0 otherwise. BMI denotes Body Mass Index, and we consider three groups according to the level of BMI. HBSC includes three age categories, and we consider them as subgroups. IRFAS represents the relative family affluence scale, and we consider two categories: low (IRFAS below 8), and high (IRFAS above 8). Life Satisfaction relies on participants' answers from 0 corresponding to the worst possible life to 10, corresponding to the best possible life and we consider below and above average life satisfaction.

**Table S1Di. Relation between body dissatisfaction and life satisfaction. Regression of life satisfaction (and self-efficacy, meaning in life and negative feelings in PISA) on gender (1), on gender and body dissatisfaction (2) and on gender, body dissatisfaction and its interaction with gender (3).**

**PISA2018 SAMPLE**

|  | | *Dependent Variable is...* | | | | | | | | | | | |  | | |
| --- | --- | --- | --- | --- | --- | --- | --- | --- | --- | --- | --- | --- | --- | --- | --- | --- |
|  | **Life**  **Satisfaction** | | **Life Satisfaction** | | **Life Satisfaction** | **Self-**  **Efficacy** | | **Self-**  **Efficacy** | | **Self-**  **Efficacy** | | | |  | |  |
|  | **(1)** | | **(2)** | | **(3)** | **(1)** | | **(2)** | | **(3)** | | | |  | |  |
| **Girl** | **-.139***** | | **-.088***** | | **-.089***** | **.00586** | | **.0546 ***** | | .0545 *** | | | |  | |  |
| (s.e) | (.007) | | (0.007) | | (0.007) | (.008) | | (0.007) | | (0.007) | | | |  | |  |
| ***BD*** |  | | **-.298 ***** | | -.257*** |  | | **-.305***** | | -.289*** | | | |  | |  |
| (s.e) |  | | (0.004) | | (0.005) |  | | (0.004) | | (0.005) | | | |  | |  |
| **Girl**BD*** |  | |  | | **-.0811***** |  | |  | | **-.0312***** | | | |  |  |  |
| (s.e) |  | |  | | (0.007) |  | |  | | (0.007) | | | |  | |  |
| Cons. | .0691*** | | .0418*** | | 0.0456*** | -.00107 | | -.0255*** | | -.0240*** | | | |  | |  |
| (s.e) | (.006) | | (0.005) | | (0.005) | (.0055) | | (0.005) | | (0.005) | | | |  | |  |
| R-sq. | 0.005 | | 0.094 | | 0.096 | 0 | | 0.095 | | 0.095 | | | |  | |  |
| Number of observations | 71,313 | | 71,313 | | 71,313 | 69,304 | | 69,304 | | 69,304 | | | |  | |  |
|  |  | |  | |  |  | | |  | | |  | |  | | |
|  |  | |  | |  |  | | |  | | |  | |  | | |
|  | *Dependent Variable is...* | | | | | | | | | | | | |  | | |
|  | **Life Meaning** | | **Life**  **Meaning** | | **Life Meaning** | **Feeling miserable** | **Feeling**  **miserable** | | | | **Feeling**  **miserable** | |  |  |  |  |
|  | **(1)** | | **(2)** | | **(3)** | **(1)** | **(2)** | | | | **(3)** | |  |  |  |  |
| Girl | **-.0328***** | | **+.0146**** | | +.0145** | **.220***** | **.187***** | | | | .188*** | |  | |  |  |
| (s.e) | (.008) | | (0.007) | | (0.007) | (.007) | (0.007) | | | | (0.007) | |  | |  |  |
| ***BD*** |  | | **-.297***** | | -.280*** |  | **.203***** | | | | .160*** | |  | |  |  |
| (s.e) |  | | (0.004) | | (0.005) |  | (0.004) | | | | (0.005) | |  | |  |  |
| **Girl**BD*** |  | |  | | **-.0337 ***** |  | | | | | **.0840***** | |  |  |  |  |
| (s.e) |  | |  | | (0.007) |  |  | | | | (0.007) | |  | |  |  |
| Cons. | .0019*** | | -.0104*** | | -.0089* | -.113*** | -.0964*** | | | | -.0999*** | |  | |  |  |
| (s.e) | (.0056) | | (0.005) | | (0.005) | (.005) | (0.005) | | | | (0.005) | |  | |  |  |
| R-sq. | 0. | | 0.088 | | 0.089 | 0.012 | 0.054 | | | | 0.056 | |  | |  |  |
| Number of observations | 69,630 | | 69,630 | | 69,630 | 69,518 | 69,518 | | | | 69,518 | |  | |  |  |
|  | |  | |  |  |  | | |  | | |  | |  | | |

|  | | *Dependent Variable is...* | | | | | | | | | | | |  | | |
| --- | --- | --- | --- | --- | --- | --- | --- | --- | --- | --- | --- | --- | --- | --- | --- | --- |
|  | **Life**  **Satisfaction** | | **Life Satisfaction** | | **Life Satisfaction** | **Self-**  **Efficacy** | | **Self-**  **Efficacy** | | **Self-**  **Efficacy** | | | |  | |  |
|  | **(1)** | | **(2)** | | **(3)** | **(1)** | | **(2)** | | **(3)** | | | |  | |  |
| **Girl** | **-.140***** | | **-.062***** | | **-.063***** | **-.00169** | | **.0666 ***** | | .0664 *** | | | |  | |  |
| (s.e) | (.008) | | (0.007) | | (0.007) | (.008) | | (0.007) | | (0.007) | | | |  | |  |
| ***WADI*** |  | | **-.300 ***** | | -.273*** |  | | **-.265***** | | -.258*** | | | |  | |  |
| (s.e) |  | | (0.004) | | (0.005) |  | | (0.004) | | (0.005) | | | |  | |  |
| **Girl**WADI*** |  | |  | | **-.0493***** |  | |  | | **-.0121** | | | |  |  |  |
| (s.e) |  | |  | | (0.007) |  | |  | | (0.007) | | | |  | |  |
| Cons. | .0726*** | | .0308*** | | 0.0345*** | .0093 * | | -.0255*** | | -.0247*** | | | |  | |  |
| (s.e) | (.005) | | (0.005) | | (0.005) | (.0055) | | (0.005) | | (0.005) | | | |  | |  |
| R-sq. | 0.005 | | 0.095 | | 0.096 | 0 | | 0.071 | | 0.071 | | | |  | |  |
| Number of observations | 68,176 | | 68,176 | | 68,176 | 66,484 | | 66,484 | | 66,484 | | | |  | |  |
|  |  | |  | |  |  | | |  | | |  | |  | | |
|  |  | |  | |  |  | | |  | | |  | |  | | |
|  | *Dependent Variable is...* | | | | | | | | | | | | |  | | |
|  | **Life Meaning** | | **Life**  **Meaning** | | **Life Meaning** | **Feeling miserable** | **Feeling**  **miserable** | | | | **Feeling**  **miserable** | |  |  |  |  |
|  | **(1)** | | **(2)** | | **(3)** | **(1)** | **(2)** | | | | **(3)** | |  |  |  |  |
| Girl | **-.0360***** | | **+.0353***** | | +.0350*** | **.225***** | **.172***** | | | | .173*** | |  | |  |  |
| (s.e) | (.008) | | (0.007) | | (0.007) | (.008) | (0.008) | | | | (0.008) | |  | |  |  |
| ***WADI*** |  | | **-.280***** | | -.269*** |  | **.206***** | | | | .164*** | |  | |  |  |
| (s.e) |  | | (0.004) | | (0.005) |  | (0.004) | | | | (0.006) | |  | |  |  |
| **Girl**WADI*** |  | |  | | **-.0213 ***** |  | | | | | **.0781***** | |  |  |  |  |
| (s.e) |  | |  | | (0.007) |  |  | | | | (0.008) | |  | |  |  |
| Cons. | .0019*** | | -.0181*** | | -.0166*** | -.118*** | -.0907*** | | | | -.0963 *** | |  | |  |  |
| (s.e) | (.0056) | | (0.005) | | (0.005) | (.006) | (0.005) | | | | (0.005) | |  | |  |  |
| R-sq. | 0. | | 0.078 | | 0.078 | 0.013 | 0.055 | | | | 0.057 | |  | |  |  |
| Number of observations | 66,743 | | 66,743 | | 66,743 | 66,613 | 66,613 | | | | 66,613 | |  | |  |  |
|  | |  | |  |  |  | | |  | | |  | |  | | |

Source: PISA2018.

Notes: The table presents the estimates of the regression of life satisfaction, self-efficacy, meaning in life and negative feelings (frequency of feeling miserable) on a dummy for gender in specification (1), on both gender and body dissatisfaction in Specification (2) and on gender, body dissatisfaction and their interaction in Specification (3). Body dissatisfaction is measured by our main indicator *BD* as well as the *Weight and Appearance Dissatisfaction Index* *WADI* (see e.g. the notes of Table S1A for the description of *BD* and *WADI*). Life Satisfaction is measured through answers to the question 'Overall, how satisfied are you with your life as a whole these days?' on a scale from 0 to 10). The variable 'Feeling miserable' is measured through answers to the question: 'how frequently (“never”, “rarely”, “sometimes”, “always”) do you feel miserable?'. Self-efficacy and meaning in life are measured by indices provided by PISA, relying on a series of items. All variables are standardized to have a weighted mean equal to 0 and a weighted standard deviation equal to 1 in each country. See Appendix A for more details on these variables. Standard errors in parentheses. **** p<0.01, ** p<0.05, * p<0.1*

**HBSC2018 SAMPLE**

|  | *Dependent Variable is...* | | |
| --- | --- | --- | --- |
|  | **Life Satisfaction**  **(1)** | **Life Satisfaction**  **(2)** | **Life Satisfaction**  **(3)** |
| **Girl** | **-.134***** | **-.098***** | -.099*** |
| (s.e) | (.004) | (0.004) | (0.004) |
| ***ThinkTooFat*** |  | **-.190***** | -.137*** |
| (s.e) |  | (0.002) | (0.003) |
| **Girl**ThinkTooFat*** |  |  | **-.0919***** |
| (s.e) |  |  | (0.004) |
| Cons. | .068*** | .050*** | .0548*** |
| (s.e) | (.003) | (0.003) | (0.003) |
| Number of observations | 226,024 | 226,024 | 226,024 |
|  |  |  |  |

Source: PISA2018

Notes: The table presents the estimates of the regression of Life satisfaction on a dummy for gender in specification (1), on both gender and body dissatisfaction in Specification (2) and on gender, body dissatisfaction and their interaction in Specification (3). Body dissatisfaction is measured by our main indicator *ThinkTooFat* (see e.g. the notes of Table S1A for the description of *ThinkTooFat*). The dependent variable is Life Satisfaction (relying on participants' answers from 0 corresponding to the worst possible life to 10, corresponding to the best possible life). All variables are standardized to have a weighted mean equal to 0 and a weighted standard deviation equal to 1 in each country. See Appendix A for more details on these variables. Standard errors in parentheses. **** p<0.01, ** p<0.05, * p<0.1*

**Table S1Dii. Relation between body dissatisfaction and life satisfaction (and self-efficacy, meaning in life, or feeling miserable in PISA): For boys and girls separately**

| **PISA2018 SAMPLE**   \|  \|  \| \|  \| \|  \|  \| \|  \|  \| \|  \|  \| \| --- \| --- \| --- \| --- \| --- \| --- \| --- \| --- \| --- \| --- \| --- \| --- \| --- \| \|  \| **Life satisfaction** \| \| \| \| **Self-efficacy** \| \|  \| **Life meaning** \| \|  \| **Feeling miserable** \| \| \|  \| **GIRL** \| \| **BOY** \| \| **GIRL** \| **BOY** \| \| **GIRL** \| **BOY** \| \| **GIRL** \| **BOY** \| \| ***BD*** \|  \| \|  \| \|  \|  \| \|  \|  \| \|  \|  \| \| R-sq. \| 0.117 \| \| 0.071 \| \| 0.113 \| 0.081 \| \| 0.104 \| 0.076 \| \| 0.066 \| 0.026 \| \| Coeff \| -0.34^***^ \| \| -0.26^***^ \| \| -0.32^***^ \| -0.29^***^ \| \| -0.32^***^ \| -0.28^***^ \| \| 0.25^***^ \| 0.16^***^ \| \|  \|  \| \|  \| \|  \|  \| \|  \|  \| \|  \|  \| \| ***Body dislike*** \| \|  \|  \| \|  \|  \| \|  \|  \| \|  \|  \| \| R-sq. \| 0.086 \| \| 0.049 \| \| 0.070 \| 0.042 \| \| 0.073 \| 0.046 \| \| 0.050 \| 0.016 \| \| Coeff. \| -0.29^***^ \| \| -0.22^***^ \| \| -0.25^***^ \| -0.21^***^ \| \| -0.26^***^ \| -0.22^***^ \| \| 0.22^***^ \| 0.13^***^ \| \|  \|  \| \|  \| \|  \|  \| \|  \|  \| \|  \|  \| \| ***Body Weight concern*** \| \| \| \|  \|  \|  \| \|  \|  \| \|  \|  \| \| R-sq. \| 0.018 \| \| 0.011 \| \| 0.015 \| 0.012 \| \| 0.017 \| 0.011 \| \| 0.014 \| 0.003 \| \| Coeff \| -0.13^***^ \| \| -0.10^***^ \| \| -0.12^***^ \| -0.11^***^ \| \| -0.13^***^ \| -0.11^***^ \| \| 0.11^***^ \| 0.06^***^ \| \|  \|  \| \|  \| \|  \|  \| \|  \|  \| \|  \|  \| \| ***Body Look Dissatisfaction*** \|  \| \|  \| \|  \|  \| \|  \|  \| \|  \|  \| \| R-sq. \| 0.136 \| \| 0.087 \| \| 0.097 \| 0.078 \| \| 0.108 \| 0.079 \| \| 0.073 \| 0.035 \| \| Coeff \| -0.37^***^ \| \| -0.29^***^ \| \| -0.30^***^ \| -0.28^***^ \| \| -0.33^***^ \| -0.29^***^ \| \| 0.27^***^ \| 0.19^***^ \| \|  \|  \| \|  \| \|  \|  \| \|  \|  \| \|  \|  \| \| ***Weight and Appearance Dissatisfaction Index*** \|  \| \|  \| \|  \|  \| \|  \|  \| \|  \|  \| \| R-sq. \| 0.11 \| \| 0.07 \| \| 0.08 \| 0.06 \| \| 0.09 \| 0.07 \| \| 0.07 \| 0.03 \| \| Coeff. \| -0.32^***^ \| \| -0.27^***^ \| \| -0.27^***^ \| -0.26^***^ \| \| -0.29^***^ \| -0.27^***^ \| \| 0.24^***^ \| 0.16^***^ \| \|  \| \| \| \| \|  \|  \| \|  \|  \| \|  \|  \|  \|  \| |
| --- | --- | --- | --- | --- | --- | --- | --- | --- | --- | --- | --- | --- | --- | --- | --- | --- | --- | --- | --- | --- | --- | --- | --- | --- | --- | --- | --- | --- | --- | --- | --- | --- | --- | --- | --- | --- | --- | --- | --- | --- | --- | --- | --- | --- | --- | --- | --- | --- | --- | --- | --- | --- | --- | --- | --- | --- | --- | --- | --- | --- | --- | --- | --- | --- | --- | --- | --- | --- | --- | --- | --- | --- | --- | --- | --- | --- | --- | --- | --- | --- | --- | --- | --- | --- | --- | --- | --- | --- | --- | --- | --- | --- | --- | --- | --- | --- | --- | --- | --- | --- | --- | --- | --- | --- | --- | --- | --- | --- | --- | --- | --- | --- | --- | --- | --- | --- | --- | --- | --- | --- | --- | --- | --- | --- | --- | --- | --- | --- | --- | --- | --- | --- | --- | --- | --- | --- | --- | --- | --- | --- | --- | --- | --- | --- | --- | --- | --- | --- | --- | --- | --- | --- | --- | --- | --- | --- | --- | --- | --- | --- | --- | --- | --- | --- | --- | --- | --- | --- | --- | --- | --- | --- | --- | --- | --- | --- | --- | --- | --- | --- | --- | --- | --- | --- | --- | --- | --- | --- | --- | --- | --- | --- | --- | --- | --- | --- | --- | --- | --- | --- | --- | --- | --- | --- | --- | --- | --- | --- | --- | --- | --- | --- | --- | --- | --- | --- | --- | --- | --- | --- | --- | --- | --- | --- | --- | --- | --- | --- | --- | --- | --- | --- | --- | --- | --- | --- | --- | --- | --- | --- | --- | --- | --- | --- | --- | --- | --- | --- | --- | --- | --- | --- | --- | --- | --- | --- | --- | --- | --- | --- | --- | --- | --- | --- | --- | --- | --- | --- | --- | --- | --- | --- | --- | --- | --- | --- | --- | --- | --- | --- | --- | --- | --- | --- | --- | --- | --- | --- | --- | --- | --- | --- | --- | --- | --- | --- | --- | --- | --- | --- | --- |

Source: PISA 2018.

Notes: The table displays the R-squared and the estimates of the coefficients of body dissatisfaction in the regression of indicators of life satisfaction, self-efficacy, meaning in life, negative feelings on indicators of body dissatisfaction, for girls and boys. Regressions are run separately for girls and for boys. We consider five different indicators of body dissatisfaction, including our main measure *BD*. See the notes of Table S1A for the descriptions of these indicators. In the first two columns, the dependent variable is Life Satisfaction (relying on answers to 'Overall, how satisfied are you with your life as a whole these days?' on a scale from 0 to 10) and explanatory variable is Body Dissatisfaction measured by the main indicator (*BD*) as well as by the four alternative variables. Other dependent variables include the variable 'Feeling miserable' (relying on answers to the question: 'how frequently (“never”, “rarely”, “sometimes”, “always”) do you feel miserable?', last two columns), as well as Self-efficacy and meaning in life, measured by indices provided by PISA, relying on a series of items. All variables are standardized to have a weighted mean equal to 0 and a weighted standard deviation equal to 1 in each country. See Appendix A for more details on these variables. ^*^ *p* < 0.10, ^**^ *p* < 0.05, ^***^ *p* < 0.01

**HBSC SAMPLE**

|  | **Life Satisfaction** | |
| --- | --- | --- |
|  | **GIRL** | **BOY** |
| ***ThinkTooFat*** | |  |
| R-sq. | 0.055 | 0.017 |
| Coeff. | -0.229*** | -0.137*** |

Source: HBSC 2018.

Notes: The table displays the R-squared and the estimates of the coefficients of body dissatisfaction in the regression of life satisfaction on body dissatisfaction, for girls and boys. Regressions are run separately for girls and for boys. The dependent variable is Life Satisfaction (relying on participants' answers from 0 corresponding to the worst possible life to 10, corresponding to the best possible life). The measure *ThinkTooFat* of Body dissatisfaction relies on one question in HBSC survey about what the participants think of their body, being equal to 1 if participants answer "too fat", to 2 if participants answer "much too fat" and to 0 otherwise. Variables are standardized to have a weighted mean equal to 0 and a weighted standard deviation equal to 1 in each country. See Appendix A for more details on these variables. ^*^ *p* < 0.10, ^**^ *p* < 0.05, ^***^ *p* < 0.01

**Table S2Ai. The gender gap in body dissatisfaction by country**

**PISA SAMPLE**

|  | ***BD*** | ***Body***  ***Dislike*** | ***Body***  ***Weight concern*** | ***Body***  ***Look Dissatisfaction*** | ***Weight and Appearance Dissatisfaction Index*** |
| --- | --- | --- | --- | --- | --- |
| **All** | 0.15*** | 0.20*** | 0.20*** | 0.17*** | 0.25^***^ |
|  |  |  |  |  |  |
| **REGIONS** |  |  |  |  |  |
| **OECD countries** | 0.36*** | 0.36*** | 0.21*** | 0.35*** | 0.38^***^ |
| **Non-OECD countries** | 0.04*** | 0.11*** | 0.19*** | 0.07*** | 0.17^***^ |
| **BY COUNTRY (N=9)** |  |  |  |  |  |
| Spain | 0.33*** | 0.33*** | 0.24*** | 0.32*** | 0.38^***^ |
| Ireland | 0.59*** | 0.53*** | 0.32*** | 0.49*** | 0.55^***^ |
| Mexico | 0.12*** | 0.18*** | 0.06** | 0.22*** | 0.18^***^ |
| UAE | -0.08** | -0.01 | 0.09*** | -0.03 | 0.04 |
| Bulgaria | 0.04 | 0.15*** | 0.21*** | 0.04 | 0.20^***^ |
| Georgia | 0.14*** | 0.20*** | 0.23*** | 0.09*** | 0.24^***^ |
| Hong Kong | 0.17*** | 0.27*** | 0.41*** | 0.19*** | 0.37^***^ |
| Panama | 0.00 | 0.00 | 0.03 | 0.08** | 0.04 |
| Serbia | -0.02 | 0.06* | 0.15*** | 0.08** | 0.15^***^ |

Source: PISA 2018.

Notes: The table presents the gender gap in body dissatisfaction by country in the PISA sample. Body dissatisfaction is measured by the main indicator *BD*, as well as by alternative variables, all described in main text and in Appendix A (also see the Notes of Table S1A). For the five variables, a positive gender gap represents a higher dissatisfaction among girls. The variables are standardized to have a weighted mean equal to 0 and a weighted standard deviation equal to 1 in each country. **** p<0.01, ** p<0.05, * p<0.1*

**HBSC SAMPLE**

|  | **HBSC**  ***ThinkTooFat*** |  |  |  |
| --- | --- | --- | --- | --- |
| **All** | 0.19^***^ |  |  |  |
|  |  |  |  |  |
| **REGIONS** |  |  |  |  |
| **OECD countries** | 0.23^***^ |  |  |  |
| **Non-OECD countries** | 0.14^***^ |  |  |  |
| **BY COUNTRY (N=41)** |  |  | |  |
| ALL | 0.192*** | |  |  |
| Albania | -0.027 | |  |  |
| Armenia | 0.001 | |  |  |
| Austria | 0.226*** | |  |  |
| Azerbaijan | 0.040 | |  |  |
| Belgium (French) | 0.380*** | |  |  |
| Bulgaria | -0.004 | |  |  |
| Canada | 0.109*** | |  |  |
| Switzerland | 0.285*** | |  |  |
| Czech Republic | 0.131*** | |  |  |
| Germany | 0.242*** | |  |  |
| Denmark | 0.367*** | |  |  |
| Estonia | 0.212*** | |  |  |
| Spain | 0.195*** | |  |  |
| France | 0.252*** | |  |  |
| England | 0.224*** | |  |  |
| Georgia | 0.031 | |  |  |
| Greenland | 0.425*** | |  |  |
| Greece | 0.205*** | |  |  |
| Croatia | 0.137*** | |  |  |
| Hungary | 0.310*** | |  |  |
| Ireland | 0.344*** | |  |  |
| Israël | 0.087*** | |  |  |
| Iceland | 0.168*** | |  |  |
| Italy | 0.179*** | |  |  |
| Kazakhstan | 0.114*** | |  |  |
| Lithuania | 0.265*** | |  |  |
| Luxembourg | 0.201*** | |  |  |
| Latvia | 0.271*** | |  |  |
| Moldova | 0.157*** | |  |  |
| Malta | -0.041 | |  |  |
| Netherlands | 0.389*** | |  |  |
| Poland | 0.325*** | |  |  |
| Portugal | 0.155*** | |  |  |
| Romania | 0.133*** | |  |  |
| Serbia | 0.045 | |  |  |
| Russia | 0.182*** | |  |  |
| Sweden | 0.376*** | |  |  |
| Slovenia | 0.238*** | |  |  |
| Slovakia | 0.122*** | |  |  |
| Turkey | 0.049* | |  |  |
| Ukraine | 0.242*** | |  |  |

Source: HBSC 2018.

Notes: The table presents the gender gap in Body Dissatisfaction by country in the HBSC sample. The measure *ThinkTooFat* of Body dissatisfaction relies on one question in HBSC survey about what the participants think of their body, being equal to 1 if participants answer "too fat", to 2 if participants answer "much too fat" and to 0 otherwise. We consider the group of countries in the sample that are OECD countries and those that are not, as well as each country separately. **** p<0.01, ** p<0.05, * p<0.1*

**Table S2Aii. Variations (standard deviation) across countries of boys' and girls' body dissatisfaction and their correlation across countries with the gender gap in body dissatisfaction.**

|  | SD  BOY | | SD  GIRL | SD  GAP | Correlation BOY  and GAP | Correlation GIRL  and GAP |
| --- | --- | --- | --- | --- | --- | --- |
| **HBSC2018 data: N=41** | |  |  |  |  |  |
| ***ThinkTooFat*** | | 0.06 | 0.11 | 0.12 | 0.30 | 0.79^***^ |
|  | |  |  |  |  |  |

| **PISA2018 data: N=9** |  | | |  |  | |  |  | |  |
| --- | --- | --- | --- | --- | --- | --- | --- | --- | --- | --- |
| ***BD*** | | 0.16 | | 0.27 | 0.21 | | 0.16 | 0.82^***^ | |  |
| ***Body Dislike*** | | 0.10 | | 0.19 | 0.17 | | 0.21 | 0.87^***^ | |  |
| ***Body Weight concern*** | | 0.15 | | 0.21 | 0.12 | | 0.30 | 0.73^**^ | |  |
| ***Body Look Dissatisfaction*** | | 0.10 | | 0.19 | 0.16 | | -0.11 | 0.65^*^ | |  |
| ***Weight and Appearance Dissatisfaction Index*** | | 0.11 | | 0.19 | 0.17 | | 0.46 | 0.86^***^ | |  |
|  | | |  |  |  | |  |  | | |
|  |  | | |  | |  |  | |  | |

Notes: The table presents the standard deviation across countries of measures of boys' body dissatisfaction (column 1), girls' body dissatisfaction (column 2) and of the gender gap in body dissatisfaction (column 3). It also displays the relation across countries between the gender gap in body dissatisfaction and boys' (column 4) and girls' (column 5) body dissatisfaction. For HBSC data, there are N=41 observations (or countries), and the measure of body dissatisfaction is provided by the variable *ThinkTooFat*. For PISA data, there are N=9 observations (or countries), and the measures of body dissatisfaction are given by the main measure *BD*, as well as the measures of *Body dislike*, *Body weight concern*, *Body Look Dissatisfaction* and the *Weight and Appearance Dissatisfaction Index*. All measures of body dissatisfaction are described in the Notes of Table S1A (see also main text and AppendixA). ^*^ *p* < 0.10, ^**^ *p* < 0.05, ^***^ *p* < 0.01

**Table S2Bi. Relations between country-level gender gaps in eating disorders, depression and life dissatisfaction and country-level gender gaps in body dissatisfaction**

|  | **Gap**  **eating disorders** | **Gap**  **depression** | **Gap**  **life dissatisfaction** |
| --- | --- | --- | --- |
| **HBSC data: N=41** |  |  |  |
| **GENDER GAP IN...** |  |  |  |
| ***ThinkTooFat*** | 0.38** | 0.31* | 0.63*** |
| ***ThinkTooFat* 15y.o.** | 0.40*** | 0.34** | 0.59*** |
|  |  |  |  |
| **PISA data: N=9** |  |  |  |
| **GENDER GAP IN...** |  |  |  |
| ***BD*** | 0.66* | 0.82** | 0.55 |
| ***Body Dislike*** | 0.62 | 0.80** | 0.54 |
| ***Body Weight Concern*** | 0.38 | 0.69* | 0.35 |
| ***Body Look Dissatisfaction*** | 0.72** | 0.81** | 0.66 |
| ***Weight and Appearance Dissatisfaction Index*** | 0.62 | 0.83** | 0.57 |
|  |  |  |  |

Notes: The table presents estimates at the country level of the coefficients of correlation between gender gaps (Girls-Boys) in life dissatisfaction (col. 3), in the prevalence of eating disorders (col. 1) and depression (col. 2) and measures of gender gaps in body dissatisfaction. Note that these estimates also correspond to the coefficients of linear regressions with standardized variables (see Appendix B). For PISA data, there are 9 observations (or countries), and the measures of body dissatisfaction are given by the main measure *BD*, as well as the measures of *General Body Dislike*, *Body Weight Concern*, *Body Look Dissatisfaction* and *the Index of Weight and Appearance Dissatisfaction*. For HBSC data, there are 40 observations (or countries), and the measure of body dissatisfaction is given by the variable *ThinkTooFat* as well as the latter variable limited to the category of 15 years old only. The body dissatisfaction measures are all defined in the Notes of Table S1A. The measures and data sources are described in detail in Appendix A. ^***^ *p<0.01,* ^**^ *p<0.05,* ^*^ *p<0.1*

**Table S2Bii. Relations between country levels of eating disorders, depression, life dissatisfaction and levels of body dissatisfaction for boys, girls and gender gaps. HBSC data.**

|  | **Gap ED** | **Girl ED** | **Boy ED** | **Gap**  **Dep** | **Girl**  **Dep** | **Boy**  **Dep** | **Gap**  **Life Dis.** | **Girl**  **Life Dis.** | **Boy**  **Life Dis.** |
| --- | --- | --- | --- | --- | --- | --- | --- | --- | --- |
| **Gap *ThinkTooFat*** | 0.38** | 0.40*** | 0.46*** | 0.31* | 0.44*** | 0.47*** | 0.63*** | 0.33** | 0.08 |
| **Girl *ThinkTooFat*** | 0.38** | 0.40*** | 0.43*** | 0.24 | 0.29* | 0.26 | 0.64*** | 0.48*** | 0.26 |
| **Boy *ThinkTooFat*** | 0.26* | 0.26* | 0.25 | 0.11 | 0.07 | 0. | 0.42*** | 0.44*** | 0.33** |
| **Gap *ThinkTooFat* 15y.o.** | 0.40*** | 0.43*** | 0.49*** | 0.34** | 0.52*** | 0.59*** | 0.59*** | 0.32** | 0.09 |
| **Girl *ThinkTooFat* 15y.o.** | 0.46*** | 0.49*** | 0.51*** | 0.34** | 0.41*** | 0.38** | 0.57*** | 0.46*** | 0.27* |
| **Boy *ThinkTooFat* 15y.o.** | 0.36** | 0.36** | 0.36** | 0.17 | 0.08 | 0.07 | 0.45*** | 0.43*** | 0.30* |

Source: HBSC2018.

Notes: The table presents the matrix of cross-country correlation between the levels of eating disorders (ED), depression (Dep), life dissatisfaction (Life Dis.) and body dissatisfaction for girls, for boys and for their differences (gender gap G-B). The data are from HBSC2018 survey with N=41, the measure of body dissatisfaction is given by the variable *ThinkTooFat* as well as the latter variable limited to the category of 15 years old only (see the Notes of Table S1A for its description). The measures and data sources are described in detail in main text and in Appendix A. ^*^ *p* < 0.10, ^**^ *p* < 0.05, ^***^ *p* < 0.01

**Table S2Biii. Cross-country correlation matrix for gender gaps across various measures of body dissatisfaction. PISA sample.**

|  | ***BD*** | ***General Body Dislike*** | ***Body Weight Concern*** | ***Body***  ***Look Dissatisfaction*** | ***WADI*** |
| --- | --- | --- | --- | --- | --- |
| ***BD*** | 1 |  |  |  |  |
| ***Body Dislike*** | 0.97*** | 1 |  |  |  |
| ***Body Weight*** | 0.61* | 0.73** | 1 |  |  |
| ***Body Look*** | 0.96*** | 0.92*** | 0.5 | 1 |  |
| ***WADI*** | 0.94*** | 0.99*** | 0.82*** | 0.89*** | 1 |
| **WB153q01** | 0.97*** | 0.91*** | 0.47 | 1 | 0.88*** |
| **WB153q02** | 0.95*** | 0.92*** | 0.7** | 0.88*** | 0.93*** |
| **WB153q05** | 0.93*** | 0.86*** | 0.35 | 0.88*** | 0.80*** |

Notes: The table shows the cross-country correlation matrix for gender gaps across various measures of body dissatisfaction. There are 9 observations (or countries), and the measures of body dissatisfaction are given by our main measure *BD*, as well as our measures for *Body dislike*, *Body weight dissatisfaction*, *Body Look dissatisfaction*, and the *Index of Weight and Appearance Dissatisfaction*, to which we add participants' answers to 'I like my look just the way it is' (WB153q01ha), 'I consider myself to be attractive' (WB153q02ha), and 'I like the way my clothes fit me' (WB153q05ha). The measures and data sources are described in detail in main text and in Appendix A. ^*^ *p* < 0.10, ^**^ *p* < 0.05, ^***^ *p* < 0.01

**Table S2C. The gender equality paradox. Country-level correlations between gender gaps in body dissatisfaction and country levels of development, wealth, gender equality and individualism**

|  | **HDI** | | **lGDP** | **GGI** | **Individualism** |
| --- | --- | --- | --- | --- | --- |
|  |  | |  |  |  |
| **HBSC data: N=41** | | |  |  |  |
| **Gap *ThinkTooFat*** | | **0.62^***^** | **0.58^***^** | **0.47^***^** | **0.61^***^** |
|  | |  |  |  |  |
|  | |  |  |  |  |
|  | |  |  |  |  |
| **PISA data: N=9** | | |  |  |  |
| **Gap *BD*** | | 0.66* | 0.52 | 0.69* | 0.83*** |
| **Gap *Body Dislike*** | | 0.66* | 0.48 | 0.70* | 0.80*** |
| **Gap *Body Weight*** | | 0.72** | 0.40 | 0.53 | 0.47 |
| **Gap *Body Look*** | | 0.61* | 0.52 | 0.74** | 0.72** |
| **Gap WADI** | | 0.72** | 0.50 | 0.72^**^ | 0.76^***^ |
|  |  | |  |  |  |

Notes: The table presents the cross-country correlation between the gender gap in various measures of body dissatisfaction and country-level measures of development, wealth, gender equality and individualism. Country development is measured by the Human Development Index (HDI), country wealth by the log of the Gross Domestic Product (lGDP), country gender equality by the General Gender Gap Index (GGI), and country individualism by Hofstede's measure of Individualism. For HBSC data, there are N=40 observations (or countries), and body dissatisfaction is measured by the variable *ThinkTooFat* which addresses concerns related to body fatness. For PISA data, there are 9 observations (or countries), and the measures of body dissatisfaction are given by the primary measure *BD*, as well as the measures of *General Body Dislike*, *Body Weight Concern*, *Body Look Dissatisfaction* and *the Index of Weight and Appearance Dissatisfaction (WADI)*. The measures and data sources are described in detail in Appendix A (see the Notes of Table S1A for the description of the measures of body dissatisfaction). ^*^ *p* < 0.10, ^**^ *p* < 0.05, ^***^ *p* < 0.01

**Table S2D. Evidence of the gender equality paradox at the micro level. HBSC sample**

|  | *Dependent Variable is body dissatisfaction (ThinkTooFat)* | | | | | |
| --- | --- | --- | --- | --- | --- | --- |
| *a) Linking the Gender gap in Body dissatisfaction to Human development (HDI)* | | | | | | |
|  |  |  |  |  |  |  |
| Girl | 0.182*** | 0.225*** | 0.181*** | 0.182*** | 0.159*** | 0.232*** |
| (s.e) | (0.015) | (0.015) | (0.015) | (0.015) | (0.014) | (0.015) |
| **Girl*HDI** | **0.074***** | **0.054***** | **0.074***** | **0.074***** | **0.062***** | **0.052***** |
| (s.e) | (0.015) | (0.014) | (0.015) | (0.015) | (0.015) | (0.014) |
| Number of observations | 199,493 | 170,214 | 198,385 | 189,530 | 196,548 | 163,549 |
| *b) Linking the Gender gap in Body dissatisfaction to Gross Domestic Product (GDP)* | | | | | | |
|  |  |  |  |  |  |  |
| Girl | 0.187*** | 0.226*** | 0.187*** | 0.187*** | 0.164*** | 0.232*** |
| (s.e) | (0.016) | (0.015) | (0.016) | (0.016) | (0.015) | (0.015) |
| **Girl*GDP** | **0.071***** | **0.045**** | **0.071***** | **0.071***** | **0.059***** | **0.043***** |
| (s.e) | (0.017) | (0.016) | (0.017) | (0.017) | (0.017) | (0.016) |
| Number of observations | 200,526 | 170,715 | 199,414 | 190,436 | 197,564 | 164,01 |
|  |  |  |  |  |  |  |
| Country fixed effects | Yes | Yes | Yes | Yes | Yes | Yes |
| Control for BMI | No | Yes | No | No | No | Yes |
| Control for age | No | No | Yes | No | No | Yes |
| Control for family affluence | No | No | No | Yes | No | Yes |
| Control for life satisfaction | No | No | No | No | Yes | No |

Notes: The table displays estimates of regressions at the individual level of body dissatisfaction, measured by the variable *ThinkTooFat*, on a dummy for gender and the dummy interacted with a measure of country level of development (Human Development Index, setting a) or level of wealth (Gross Domestic Product, setting b) with or without additional controls at the individual level. Country fixed effects are included in all specifications. The first column includes no control. Control for body mass index is included in the second column, control for age category in the third column, control for family affluence (IRFAS) in the fourth column, control for life satisfaction in the fifth column, and control for all variables but life satisfaction in the last column. See the data section in Appendix A of this SM for details about the sources for country-level measures of development or wealth and about all controls. Standard errors have been clustered at the country level. Regressions are weighted by "senate" weights which sum to one in each country. See the method section in Appendix B of this SM for details on the empirical models. *** p<0.01, ** p<0.05, * p<0.1.

**Table S2E. Country-level correlations between boys' and girls' levels of body dissatisfaction and country levels of economic development, gender equality and individualism**

|  |  |  |  |  |
| --- | --- | --- | --- | --- |
|  | **HDI** | **lGDP** | **GGI** | **Individualism** |
|  |  |  |  |  |
| **HBSC data: N=41** |  |  |  |  |
|  |  |  |  |  |
| Boy *ThinkTooFat* | 0.47^***^ | 0.39^**^ | 0.12 | 0.43^***^ |
| Girl *ThinkTooFat* | 0.65^***^ | 0.58^***^ | 0.34^**^ | 0.63^***^ |
|  |  |  |  |  |
| **PISA data: N=9** |  |  |  |  |
| Boy *BD* | 0.23 | -0.04 | 0.22 | 0.09 |
| Girl *BD* | 0.60^*^ | 0.34 | 0.65^*^ | 0.65^**^ |
| Boy *Body Dislike* | 0.32 | 0.12 | 0.05 | 0.12 |
| Girl *Body Dislike* | 0.66^*^ | 0.41 | 0.60 | 0.67^**^ |
| Boy *Body Weight* | 0.33 | 0.32 | 0.43 | -0.03 |
| Girl *Body Weight* | 0.62^*^ | 0.45 | 0.61 | 0.23 |
| Boy *Body Look* | 0.31 | 0.10 | 0.06 | 0.09 |
| Girl *Body Look* | 0.65^*^ | 0.41 | 0.60 | 0.67^**^ |
| Boy *WADI* | 0.44 | 0.23 | 0.51 | 0.07 |
| Girl *WADI* | 0.68** | 0.43 | 0.75^**^ | 0.50 |
|  |  |  |  |  |

Notes: The table presents the cross-country correlation between measures of boys' and girl's body dissatisfaction separately and country-level measures of development, wealth, gender equality and individualism. Country development is measured by the Human Development Index (HDI), country wealth by the log of the Gross Domestic Product (lGDP), country gender equality by the General Gender Gap Index (GGI), and country individualism by Hofstede's measure of Individualism. For HBSC data, there are N=40 observations (or countries), and the measures of body dissatisfaction are provided by our main variable *ThinkTooFat*, which addresses concerns related to body fatness. For PISA data, there are 9 observations (or countries), and the measures of body dissatisfaction are given by the main measure *BD*, as well as the measures of *General Body Dislike*, *Body Weight Concern*, *Body Look Dissatisfaction* and the *Index of Weight and Appearance Dissatisfaction (WADI)*. The measures and data sources are described in detail in Appendix A (see the Notes of Table S1A for the description of the measures of body dissatisfaction). ^*^ *p* < 0.10, ^**^ *p* < 0.05, ^***^ *p* < 0.01

**Table S3Ai. Relations between country levels of stereotypes**

|  | **GTS** | **IAT Career Family (Males)** | **Body-Beauty vs Talent-Strength** | **Beauty vs Talent-Strength** |  |
| --- | --- | --- | --- | --- | --- |
| **Gender Talent Stereotypes** | 1 |  |  |  |  |
| **IAT Career-Family (Males)** | 0.503^***^ | 1 |  |  |  |
| **Gender Body-Beauty vs. Talent-Strength Stereotypes** | 0.491^***^ | 0.348^**^ | 1 |  |  |
| **Gender Beauty vs. Talent-Strength Stereotypes** | 0.411^***^ | 0.484^***^ | 0.847^***^ | 1 |  |

Notes: The table presents the matrix of cross-country correlation between different gender stereotypes. We consider gender stereotypes about boys' greater talent or brilliance relying on (1) (first row), males' implicit stereotypes associating men with career and women with family, relying on Project Implicit and the Implicit Association Test (IAT), as reported in (2) (second row), and our measures of the association of beauty and body vs. talent and strength to women relative to men, relying on word embeddings pretrained on large text corpora (third and fourth rows). All the variables and data sources are described in Appendix A. ^***^ *p<0.01,* ^**^ *p<0.05,* ^*^ *p<0.1*

**Table S3Aii. Relation between country level of gender stereotypes and country level of economic development, gender equality and individualism.**

|  |  | |  |  |  |  |
| --- | --- | --- | --- | --- | --- | --- |
|  | | **HDI** | **lGDP** | **GGI** | **Individualism** | |
| **Gender Talent Stereotypes** | | 0.668^***^ | 0.554^***^ | 0.651^***^ | 0.674^***^ | |
| **IAT Career-Family (Males)** | | 0.542^***^ | 0.544^***^ | 0.203 | 0.467^***^ | |
| **Explicit career (Females)** | | 0.261^**^ | 0.341^***^ | -0.09 | 0.412^***^ | |
| **SEXY, WB** | | 0.681^***^ | 0.632^***^ | 0.345^*^ | 0.292 | |
| **Gender Body-Beauty vs. Talent-Strength Stereotypes** | | 0.362^***^ | 0.454^***^ | 0.286^**^ | 0.366^***^ | |
| **Gender Beauty vs. Talent Stereotypes** | | 0.382^***^ | 0.435^***^ | 0.203 | 0.299^**^ | |
| **Gender Beauty vs. Strength Stereotypes** | | 0.278^**^ | 0.361^***^ | 0.231^*^ | 0.344^***^ | |
| **Gender Beauty vs. Talent/Strength Stereotypes** | | 0.375^***^ | 0.452^***^ | 0.249^**^ | 0.361^***^ | |

Notes: The table presents estimates at the country level of linear regressions of gender stereotypes on country level measures of economic development. Country level of economic development is measured by the Human Development Index (HDI, column 1) and by the log of the Gross Domestic Product (lGDP, column 2), country gender equality by the General Gender Gap Index (GGI, column 3), and country individualism by Hofstede's measure of Individualism (column 4). Gender stereotypes are measured by gender stereotypes about boys greater talent relying on (1) (first row), implicit gender stereotypes about career and family for males (second row) and explicit stereotypes about career for females (third row), relying on Project Implicit, as reported in (2), the stereotype associating the adjective Sexy with women relying on (4) (fourth row), and finally our measures of the association of body and beauty vs. talent and strength to women relative to men, relying on word embeddings pretrained on large text corpora (last rows). All the variables and data sources are described in Appendix A. All variables are standardized on the regression sample. ^***^ *p<0.01,* ^**^ *p<0.05,* ^*^ *p<0.1*

**Table S3Bi. Relations between country levels of gender gaps in body dissatisfaction and country levels of gender stereotypes**

**PANEL A: PISA, N=9**

|  | *Gender Gap in...* | | | | | |  |
| --- | --- | --- | --- | --- | --- | --- | --- |
|  | ***BD*** | ***Body Dislike*** | ***Body Weight Concern*** | ***Body Look Dissatisfaction*** | ***WADI*** |  |  |
| **Gender Talent Stereotypes** | 0.791^**^ | 0.881^***^ | 0.906^***^ | 0.683^*^ | 0.931^***^ |  |  |
| **IAT Career-Family (Males)** | 0.686 | 0.759 | 0.968^***^ | 0.602 | 0.850^*^ |  |  |
| **Beauty vs Talent** | -0.22 | -0.3 | -0.71* | -0.25 | -0,46 |  |  |
| **Beauty vs Strength** | 0.62 | 0.56 | 0.01 | 0.46 | 0.43 |  |  |
| **Body-Beauty/Talent-Strength** | 0.59 | 0.51 | -0.15 | 0.45 | 0.37 |  |  |

**PANEL B: HBSC N=41**

|  | ***ThinkTooFat*** | | |
| --- | --- | --- | --- |
|  | GAP | GIRL | BOY |
| **Gender Talent Stereotypes** | 0.73*** | 0.61*** | 0.26 |
| **IAT Career-Family (Males)** | 0.47** | 0.56*** | 0.34 |
| **Explicit Career (Females)** | 0.50*** | 0.62*** | 0.41** |
| **Beauty vs Talent** | 0.29* | 0.32* | 0.24 |
| **Beauty vs Strength** | 0.43** | 0.24 | -0.02 |
| **Beauty/Talent-Strength** | 0.43** | 0.31* | 0.11 |
| **Body-Beauty/Talent-Strength** | 0.50*** | 0.24 | -0.06 |

Notes: The table presents estimates at the country level of linear regressions of gender gaps (Girls-Boys) in Body Dissatisfaction on indicators of country level of gender stereotypes. Panel A relies on PISA data, the measures of body dissatisfaction are given by the main measure *BD*, as well as the measures of *General Body Dislike*, *Body Weight Concern*, *Body Look Dissatisfaction*, and the *Index of Weight and Appearance Dissatisfaction (WADI)* and the measures of gender stereotypes include gender stereotypes about boys' greater talent or brilliance, relying on (1) (first row), implicit gender stereotypes about career and family reported by males, relying on Project Implicit, as reported in (2) (second row), and our measures of the association of body and beauty vs. talent and strength to women relative to men, relying on word embeddings pretrained on large text corpora. Panel B relies on HBSC data, and the measure of body dissatisfaction is given by our main variable *ThinkTooFat*, which addresses concerns related to body fatness. The measures of stereotypes include those of Panel A as well as females' explicit association of career with men vs. women relying on Project Implicit, as reported in (2) (third row). Panel B also provides the estimates of linear regressions of girls' and boys' body dissatisfaction on gender stereotypes. All the variables and data sources are described in Appendix A. All variables are standardized on the regression sample. Standard errors in parentheses ^***^ *p<0.01,* ^**^ *p<0.05,* ^*^ *p<0.1*

**Table S3Bii. Gender gaps in body dissatisfaction, country development and gender stereotypes. HBSC survey.**

**PANEL A: Gender Talent Stereotypes**

|  |  |  |  |  |  |
| --- | --- | --- | --- | --- | --- |
|  | *Dependent Variable is*  *Gender Gap in Body dissatisfaction (ThinkTooFat)* | | | | |
|  | **(1)** | **(2)** | **(3)** | **(4)** | **(5)** |
|  |  |  |  |  |  |
| **Gender Talent Stereotypes** | 0.731*** |  |  | 0.635*** | 0.669*** |
|  | (0.117) |  |  | (0.175) | (0.156) |
| **HDI** |  | 0.600*** |  | 0.130 |  |
|  |  | (0.137) |  | (0.175) |  |
| **lGDP** |  |  | 0.532*** |  | 0.095 |
|  |  |  | (0.145) |  | (0.156) |
|  |  |  |  |  |  |
| Constant | 3.13e-09 | 3.13e-09 | 4.73e-09 | 2.96e-09 | 3.23e-09 |
|  | (0.115) | (0.135) | (0.143) | (0.116) | (0.116) |
|  |  |  |  |  |  |
| Observations | 36 | 36 | 36 | 36 | 36 |
| R-squared | 0.535 | 0.360 | 0.283 | 0.542 | 0.540 |

**PANEL B: Gender Stereotypes about Body and Beauty relying on large text corpora**

|  | *Dependent Variable is*  *Gender Gap in Body dissatisfaction (ThinkTooFat)* | | | | |
| --- | --- | --- | --- | --- | --- |
|  | **(1)** | **(2)** | **(3)** | **(4)** | **(5)** |
|  |  |  |  |  |  |
| **Body-Beauty/Talent-Strength** | 0.504*** |  |  | 0.352** | 0.333** |
|  | (0.153) |  |  | (0.134) | (0.150) |
| **HDI** |  | 0.617*** |  | 0.513*** |  |
|  |  | (0.139) |  | (0.134) |  |
| **lGDP** |  |  | 0.565*** |  | 0.434*** |
|  |  |  | (0.146) |  | (0.150) |
|  |  |  |  |  |  |
| Constant | 5.64e-09 | 3.45e-09 | 3.44e-09 | 3.91e-09 | 4.02e-09 |
|  | (0.150) | (0.137) | (0.144) | (0.126) | (0.136) |
|  |  |  |  |  |  |
| Observations | 34 | 34 | 34 | 34 | 34 |
| R-squared | 0.254 | 0.381 | 0.319 | 0.494 | 0.413 |

Source: HBSC2018

Notes: The table presents estimates at the country level of linear regressions of the country-level gender gap (Girls-Boys) in body dissatisfaction on indicators of country level of gender stereotypes (first row) and indicators of country economic development. Body dissatisfaction is measured by the variable *ThinkTooFat* (see the description in the Notes of Table S1A), and country economic development is measured by the Human Development Index (HDI, second row) and by the log of the Gross Domestic Product (lGDP, third row). Panel A considers gender stereotypes about boys' greater talent as in (1) and Panel B considers our measure of gender stereotypes about the association of beauty/body vs. Talent/strength to women relative to men in large text corpora (see Appendix A for details about our measures). The first three columns consider single linear regression on gender stereotypes (column 1), on HDI (column 2) and on lGDP (column 3). The last two columns consider the regression of the gender gap in body dissatisfaction on gender stereotypes, controlling for HDI (column 4) and for lGDP (column 5). All the variables and data sources are described in Appendix A. All variables are standardized on the regression sample. Standard errors in parentheses ^***^ *p<0.01,* ^**^ *p<0.05,* ^*^ *p<0.1*

**Table S3Biii. Girls only. Relations between girls' body dissatisfaction and country levels of stereotypes with control for economic development. HBSC survey.**

|  | *Dependent Variable is Girls' Body dissatisfaction (ThinkTooFat)* | | | | |
| --- | --- | --- | --- | --- | --- |
|  | **(1)** | **(2)** | **(3)** | **(4)** | **(5)** |
| **Gender Talent Stereotypes** | 0.610*** |  |  | 0.375* | 0.480** |
|  | (0.136) |  |  | (0.198) | (0.179) |
| **HDI** |  | 0.595*** |  | 0.317 |  |
|  |  | (0.138) |  | (0.198) |  |
| **lGDP** |  |  | 0.512*** |  | 0.199 |
|  |  |  | (0.147) |  | (0.179) |
|  |  |  |  |  |  |
|  |  |  |  |  |  |
|  |  |  |  |  |  |
| Constant | -3.32e-10 | -6.59e-10 | 9.47e-10 | -7.60e-10 | -1.34e-10 |
|  | (0.134) | (0.136) | (0.145) | (0.131) | (0.134) |
|  |  |  |  |  |  |
| Observations | 36 | 36 | 36 | 36 | 36 |
| R-squared | 0.372 | 0.353 | 0.262 | 0.417 | 0.394 |

Notes: The table is the analog of Table S3Bii Panel A replacing the gender gap in body dissatisfaction by girls' level of body dissatisfaction. It presents estimates at the country level of linear regressions of girls' body dissatisfaction on indicators of country level of gender stereotypes about talent and indicators of country economic development. Body dissatisfaction is measured by the variable *ThinkTooFat*, gender stereotypes are measured by stereotypes about boys greater talent as in (1) and country economic development is measured by the Human Development Index (HDI) and by the log of the Gross Domestic Product (lGDP). The first three columns consider single linear regression on gender stereotypes about talent (column 1), on HDI (column 2) and on lGDP (column 3). The last two columns consider the regression of girls' body dissatisfaction on Gender Talent Stereotypes, controlling for HDI (column 4) and for lGDP (column 5). All the variables and data sources are described in Appendix A. All variables are standardized on the regression sample. Standard errors in parentheses ^***^ *p<0.01,* ^**^ *p<0.05,* ^*^ *p<0.1*

**References**

1. C. Napp, T. Breda, The stereotype that girls lack talent: A worldwide investigation. Science Advances **8**, eabm3689 (2022).

2. C. Napp, Gender Stereotypes About Career and Family Are Stronger in More Economically Developed Countries and Can Explain the Gender Equality Paradox. Personality and Social Psychology Bulletin (2024). https://doi.org/10.1177/01461672241286084.

3. B. A. Nosek, M. R. Banaji, A. G. Greenwald, Harvesting implicit group attitudes and beliefs from a demonstration web site. Group Dynamics: Theory, Research, and Practice **6**, 101–115 (2002).

4. J. E. Williams, D. L. Best, Measuring sex stereotypes: A multination study, Rev. ed (Sage Publications, Inc, 1990).

5. D. Best, J. E. Williams, Measuring Sex Stereotypes: A Multination Study. undefined (1982).

6. A. Caliskan, J. J. Bryson, A. Narayanan, Semantics derived automatically from language corpora contain human-like biases. Science **356**, 183–186 (2017).

7. C. Napp, Gender stereotypes embedded in natural language are stronger in more economically developed and individualistic countries. PNAS Nexus **2**, pgad355 (2023).

8. P. Bojanowski, E. Grave, A. Joulin, T. Mikolov, Enriching Word Vectors with Subword Information. [Preprint] (2017). Available at: http://arxiv.org/abs/1607.04606 [Accessed 25 March 2023].

9. A. Joulin, E. Grave, P. Bojanowski, T. Mikolov, Bag of Tricks for Efficient Text Classification. [Preprint] (2016). Available at: http://arxiv.org/abs/1607.01759 [Accessed 25 March 2023].

1. Instructions indicate that respondents should report the characteristics generally said to be associated with women and men in [their] culture, independent of their personal beliefs and independent of their approval of the assignment of different characteristics to men and women ((4), p51). Thus, responses should reflect general cultural stereotypes. [↑](#footnote-ref-1)
